# Supplementary material for: Unveiling the wealth-bone connection: How socioeconomic status influences trabecular bone health
Source: PLoS One. 2025 Dec 12;20(12):e0338378. doi: 10.1371/journal.pone.0338378 (PMC12700419; doi:10.1371/journal.pone.0338378)
Supplement: Attachment — Submitted filename: PONE-D-24-55944_R1 reviewed.pdf [file pone.0338378.s002.pdf]

PLOS ONE

Unveiling the Wealth-Bone Connection: How Socioeconomic Status Influences Trabecular Bone Health

--Manuscript Draft--

**Manuscript Number:**	PONE-D-24-55944R1
**Article Type:**	Research Article
**Full Title:**	Unveiling the Wealth-Bone Connection: How Socioeconomic Status Influences Trabecular Bone Health
**Short Title:**	A cross-sectional study
**Corresponding Author:**	Qice Sun<br>zhejiangsheng xinhua yiyuan: The Second Affiliated Hospital of Zhejiang Chinese Medical University<br>Hangzhou, CHINA
**Keywords:**	trabecular bone score; NHANES; Poverty income ratio; Osteoporosis; socioeconomic status
**Abstract:**	Background<br>Currently, the relationship between socioeconomic status (SES) and trabecular bone score (TBS) remains unclear. This study uses data from the National Health and Nutrition Examination Survey (NHANES) to explore the connection between SES (poverty income ratio, PIR) and TBS among American adults.<br><br>Methods<br>This study analyzed the relationship between SES and TBS in 7,832 adults using data from the 2005-2008 NHANES. TBS was measured by assessing grayscale variations in lumbar spine dual-energy X-ray absorptiometry (DXA) scans. The study employed a multivariable linear regression model, adjusting for various confounding factors, to examine the association between the PIR and TBS. Additionally, demographic characteristics were described, stratified analyses, single factor analysis, smooth curve fitting, and interaction analysis were conducted.<br><br>Results<br>Using a multivariate linear regression model, after adjusting for confounding factors, the association between SES and TBS was analyzed. Results showed that PIR was positively correlated with TBS (β = 0.0019, 95% CI 0.0002–0.0035, P < 0.05), and high-income individuals had a TBS 0.018 higher than low-income individuals (95% CI 0.0106–0.0254, P < 0.001). Smooth curve fitting analysis revealed a linear relationship between PIR and TBS, and stratified analysis showed that this positive correlation was particularly evident in Mexican Americans, non-Hispanic whites and non-Hispanic blacks aged 50 and older.<br><br>Conclusions<br>Our research shows that there is a significant positive correlation between adults' SES and TBS. Higher household income levels usually correspond to better TBS levels, which can help prevent osteoporosis and fractures. When developing strategies for the prevention and treatment of osteoporosis, healthcare professionals and policymakers should consider the impact of socioeconomic inequalities.
**Order of Authors:**	Yuan Zhang
	Tingxiao Zhao
	Yanlei Li
	Kai Chen
	Qice Sun
**Response to Reviewers:**	Dear Editors and Reviewers:<br><br>Thank you for your letter and the valuable comments from the reviewers. Based on

your constructive suggestions, we have revisited and revised the manuscript. These comments provide important guidance for improving the quality of the paper. All modifications and additions have been marked in blue font. We sincerely appreciate your positive evaluation and suggestions, and
hope that the revised manuscript will be accepted for publication in PLOS ONE.

Editor’s comments：
Thank you for submitting your manuscript to PLOS ONE. After careful consideration, we feel that it has merit but does not fully meet PLOS ONE’s publication criteria as it currently stands. Therefore, we invite you to submit a revised version of the manuscript that addresses the points raised during the review process.
Answer: Thank you for your affirmation and recognition of our article. We are fully aware that the manuscript still has shortcomings, and your professional comments are extremely valuable for improving the quality of the paper. We have made comprehensive revisions based on your suggestions and look forward to your further review and approval.

Journal Requirements:
1.Please ensure that your manuscript meets PLOS ONE's style requirements, including those for file naming. The PLOS ONE style templates can be found at
https://journals.plos.org/plosone/s/file?id=wjVg/PLOSOne_formatting_sample_main_bo

dy.pdf and
https://journals.plos.org/plosone/s/file?id=ba62/PLOSOne_formatting_sample_title_authors_affiliations.pdf
Answer: Thank you for your comments. This task has been completed according to your requirements.
2.We noticed you have some minor occurrence of overlapping text with the following previous publication(s), which needs to be addressed:
https://www.frontiersin.org/journals/endocrinology/articles/10.3389/fendo.2022.857110/full
https://pubmed.ncbi.nlm.nih.gov/36626464/
https://link.springer.com/article/10.1007/s00198-005-1917-1?
In your revision ensure you cite all your sources (including your own works), and quote or rephrase any duplicated text outside the methods section. Further consideration is dependent on these concerns being addressed.
Answer: Thank you for your comments. Your suggestions are very important for improving the quality of the manuscript. We sincerely apologize for the text overlap issue and have thoroughly revised the manuscript.

3.Please note that your Data Availability Statement is currently missing the DOI/accession number of each dataset or a direct link to access each database. If your manuscript is accepted for publication, you will be asked to provide these details on a very short timeline. We therefore suggest that you provide this information now, though we will not hold up the peer review process if you are unable.
Answer: Thank you for your comments. Your suggestions are very important for improving the quality of the manuscript. We have addressed your comments and completed the task as per your request.

4. PLOS requires an ORCID iD for the corresponding author in Editorial Manager on papers submitted after December 6th, 2016. Please ensure that you have an ORCID iD and that it is validated in Editorial Manager. To do this, go to ‘Update my Information’ (in the upper left-hand corner of the main menu), and click on the Fetch/Validate link next to the ORCID field. This will take you to the ORCID site and allow you to create a new iD or authenticate a pre-existing iD in Editorial Manager.
Answer: Thank you for your comments. This task has been completed according to your requirements.

5.Please include a separate caption for each figure in your manuscript.
Answer: Thank you for your comments. This task has been completed according to your requirements.

Additional Editor Comments:
Ensure all abbreviations are defined at first use.
Answer: Thank you for your comments. This task has been completed according to your requirements.

Introduction
Change “This study addresses the gap in ...general population”
to "This study aims to address the gap in ... general population.”
Answer: Thank you for your comments. Your suggestions are very important for improving the quality of the manuscript. We have addressed your comments and completed the task as per your request.

Fig 1 should be part of the methods. Remove arrows from figure. Use neutral connectors instead.
Answer: Thank you for your comments. Your suggestions are very important for improving the quality of the manuscript. Arrows, as internationally recognized symbols in flowcharts, are widely used in academic journal flowcharts. They visually demonstrate the sequence and direction of screening steps, conforming to international guideline standards and readers' reading habits, thereby enhancing the readability and academic quality of diagrams.
Methods
Provide the rationale for the selection of confounders and the choice statistical methods, especially for stratified and interaction analyses.

Answer: We thank the reviewer for this comment. We have added a clear rationale for our selection of confounders and statistical methods in the Methods section.

Results
Table 3 is too crowded; please divide it into two separate tables to improve clarity.
Answer: Thank you for your comments. Your suggestions are very important for improving the quality of the manuscript. We have addressed your comments and completed the task as per your request.

Discussion
Discuss the clinical significance of the small observed effect size?
Answer: Thank you for your comments. We acknowledge the importance of addressing the clinical significance of the observed effect size. We have added a new paragraph in the Discussion section to specifically address this point.

Discuss possible mechanisms linking SES and TBS.
Answer: Thank you for your comments. Your suggestions are very important for improving the quality of the manuscript. We have addressed your comments and completed the task as per your request.

Explicitly mention study limitations,....use "temporal" instead of "causal"
Answer: Thank you for your comments. This task has been completed according to your requirements.

Ethics
Add a brief statement clarifying that no ethics approval was needed due to the use of publicly available, de-identified NHANES data. Please check journal's guidelines
Answer: Thank you for your comments. This task has been completed according to your requirements.

Comments to the Author
1. Is the manuscript technically sound, and do the data support the conclusions?
The manuscript must describe a technically sound piece of scientific research with data that supports the conclusions. Experiments must have been conducted rigorously, with appropriate controls, replication, and sample sizes. The conclusions must be drawn appropriately based on the data presented.
Reviewer #1: Yes
Answer: Thank you for your comments. We appreciate your positive remarks on our manuscript. We sincerely hope that our article will be accepted for publication.

2. Has the statistical analysis been performed appropriately and rigorously?
Reviewer #1: Yes
Answer: Thank you for your comments. We appreciate your positive remarks on our manuscript. We sincerely hope that our article will be accepted for publication.

3. Have the authors made all data underlying the findings in their manuscript fully available?

The PLOS Data policy requires authors to make all data underlying the findings described in their manuscript fully available without restriction, with rare exception (please refer to the Data Availability Statement in the manuscript PDF file). The data should be provided as part of the manuscript or its supporting information, or deposited to a public repository. For example, in addition to summary statistics, the data points behind means, medians and variance measures should be available. If there are restrictions on publicly sharing data—e.g. participant privacy or use of data from a third party—those must be specified.
Reviewer #1: Yes
Answer: Thank you for your comments. We appreciate your positive remarks on our manuscript. We sincerely hope that our article will be accepted for publication.
4. Is the manuscript presented in an intelligible fashion and written in standard English?

	PLOS ONE does not copyedit accepted manuscripts, so the language in submitted articles must be clear, correct, and unambiguous. Any typographical or grammatical errors should be corrected at revision, so please note any specific errors here.<br>Reviewer #1: Yes<br>Answer: Thank you for your comments. We appreciate your positive remarks on our manuscript. We sincerely hope that our article will be accepted for publication.<br><br>6.Review Comments to the Author<br><br>Please use the space provided to explain your answers to the questions above. You may also include additional comments for the author, including concerns about dual publication, research ethics, or publication ethics. (Please upload your review as an attachment if it exceeds 20,000 characters)<br>Answer: Thank you again for your positive and constructive comments and suggestions on our manuscript. We hope you will find our revised manuscript acceptable for publication.<br><br>Reviewer #1: I don't have a lot of concerns about this study – large sample size and an unsurprising finding demonstrating a relationship between SES and TBS. There are these three things I suggest the authors correct, including:<br>Answer: We feel great thanks for your professional review work on our article. As you are concerned, there are several problems that need to be addressed. According to your nice suggestions, we have made extensive corrections to our previous draft, the detailed corrections are listed below.<br><br>1. Introduction needs a hypothesis.<br>Answer: Thank you for your comments. Your suggestions are very important for improving the quality of the manuscript. We have addressed your comments and completed the task as per your request.<br><br>2. Introduction needs expanding to include literature about other studies have looked at the relationship between SES and osteoporosis/bone health. There are plenty of them and the readers will need to get a good picture of what has already been done in this space, before understanding that there is a need to look at TBS. In fact, there have been studies that found inconsistent relationships between SES and different measures of bone remodelling/BMD etc. It will be important to establish a good overview of the current state of the literature there.<br>Answer: Thank you for your comments. Your suggestions are very important for improving the quality of the manuscript. We have addressed your comments and completed the task as per your request.<br><br>3. In Figures 2 and 3, the font on the x and y axes and in the legend, is too small.<br>Answer: Thank you for your comments. This task has been completed according to your requirements.<br><br>6. PLOS authors have the option to publish the peer review history of their article (what does this mean?). If published, this will include your full peer review and any attached files.<br>If you choose "no", your identity will remain anonymous but your review may still be made public.<br>Do you want your identity to be public for this peer review? For information about this choice, including consent withdrawal, please see our Privacy Policy.<br>Reviewer #1: No<br>We sincerely thank the editor and all reviewers for their valuable feedback that we have used to improve the quality of our manuscript. We hope our revisions meet your approval and contribute meaningful insights to the field. We welcome any further comments or suggestions.<br><br>Yours sincerely<br>Qice Sun<br>July 15, 2025
**Additional Information:**	

Question	Response
**Financial Disclosure**<br><br>Enter a financial disclosure statement that describes the sources of funding for the work included in this submission. Review the submission guidelines for detailed requirements. View published research articles from *PLOS ONE* for specific examples.<br><br>This statement is required for submission and **will appear in the published article** if the submission is accepted. Please make sure it is accurate.<br><br>**Funded studies**<br>Enter a statement with the following details:<br>• Initials of the authors who received each award<br>• Grant numbers awarded to each author<br>• The full name of each funder<br>• URL of each funder website<br>• Did the sponsors or funders play any role in the study design, data collection and analysis, decision to publish, or preparation of the manuscript?<br><br>Did you receive funding for this work?	The author(s) received no specific funding for this work.
**Competing Interests**<br><br>Use the instructions below to enter a competing interest statement for this submission. On behalf of all authors, disclose any competing interests that could be perceived to bias this work—acknowledging all financial support and any other relevant financial or non-financial competing interests.<br><br>This statement is required for submission and **will appear in the published article** if the submission is accepted. Please make sure it is accurate and that any funding sources listed in your Funding Information later in the submission form are also	The authors have declared that no competing interests exist.

declared in your Financial Disclosure statement.<br><br>View published research articles from *PLOS ONE* for specific examples.<br><br>**NO authors have competing interests**<br><br>Enter: *The authors have declared that no competing interests exist.*<br><br>**Authors with competing interests**<br><br>Enter competing interest details beginning with this statement:<br><br>*I have read the journal's policy and the authors of this manuscript have the following competing interests: [insert competing interests here]*<br><br>* typeset	
**Ethics Statement**<br><br>Enter an ethics statement for this submission. This statement is required if the study involved:<br><br>• Human participants<br>• Human specimens or tissue<br>• Vertebrate animals or cephalopods<br>• Vertebrate embryos or tissues<br>• Field research<br><br>Write "N/A" if the submission does not require an ethics statement.<br><br>General guidance is provided below. Consult the submission guidelines for detailed instructions. **Make sure that all information entered here is included in the Methods section of the manuscript.**	N/A

**Format for specific study types**<br><br>**Human Subject Research (involving human participants and/or tissue)**<br>• Give the name of the institutional review board or ethics committee that approved the study<br>• Include the approval number and/or a statement indicating approval of this research<br>• Indicate the form of consent obtained (written/oral) or the reason that consent was not obtained (e.g. the data were analyzed anonymously)<br><br>**Animal Research (involving vertebrate animals, embryos or tissues)**<br>• Provide the name of the Institutional Animal Care and Use Committee (IACUC) or other relevant ethics board that reviewed the study protocol, and indicate whether they approved this research or granted a formal waiver of ethical approval<br>• Include an approval number if one was obtained<br>• If the study involved *non-human primates*, add *additional details* about animal welfare and steps taken to ameliorate suffering<br>• If anesthesia, euthanasia, or any kind of animal sacrifice is part of the study, include briefly which substances and/or methods were applied<br><br>**Field Research**<br><br>Include the following details if this study involves the collection of plant, animal, or other materials from a natural setting:<br>• Field permit number<br>• Name of the institution or relevant body that granted permission	
**Data Availability**<br><br>Authors are required to make all data underlying the findings described fully available, without restriction, and from the time of publication. PLOS allows rare exceptions to address legal and ethical concerns. See the PLOS Data Policy and FAQ for detailed information.	Yes - all data are fully available without restriction

A Data Availability Statement describing where the data can be found is required at submission. Your answers to this question constitute the Data Availability Statement and **will be published in the article**, if accepted.<br><br>**Important:** Stating 'data available on request from the author' is not sufficient. If your data are only available upon request, select 'No' for the first question and explain your exceptional situation in the text box.<br><br>Do the authors confirm that all data underlying the findings described in their manuscript are fully available without restriction?	
**Describe where the data may be found in full sentences. If you are copying our sample text, replace any instances of XXX with the appropriate details.**<br><br>• If the data are **held or will be held in a public repository**, include URLs, accession numbers or DOIs. If this information will only be available after acceptance, indicate this by ticking the box below. For example: *All XXX files are available from the XXX database (accession number(s) XXX, XXX.).*<br>• If the data are all contained **within the manuscript and/or Supporting Information files**, enter the following: *All relevant data are within the manuscript and its Supporting Information files.*<br>• If neither of these applies but you are able to provide **details of access elsewhere**, with or without limitations, please do so. For example:<br><br>*Data cannot be shared publicly because of [XXX]. Data are available from the XXX Institutional Data Access / Ethics Committee (contact via XXX) for researchers who meet the criteria for access to confidential data.*<br><br>*The data underlying the results presented in the study are available from (include the name of the third party*	This study utilized the publicly available dataset from the NHANES in the United States (http://www.cdc.gov/nchs/nhanes/). The data were anonymized prior to analysis, participants provided informed consent, and approval was obtained from the Institutional Review Board of the National Center for Health Statistics.

and contact information or URL).<br>• This text is appropriate if the data are owned by a third party and authors do not have permission to share the data.<br><br>* typeset	
Additional data availability information:	

Manuscript Click here to access/download;Manuscript;Manuscript.docx

Title of Manuscript:

Unveiling the Wealth-Bone Connection: How Socioeconomic Status Influences Trabecular Bone Health

Author List:

Yuan Zhang[1¶], Tingxiao Zhao[2¶], Yanlei Li[2], Kai Chen[1*], Qice Sun[1*].

Affiliations:

1. Department of Rheumatology, The Second Affiliated Hospital of Zhejiang Chinese Medical University, Hangzhou, China.

2. Center for Plastic & Reconstructive Surgery, Department of Orthopedics, Zhejiang Provincial People's Hospital (Affiliated People's Hospital, Hangzhou Medical College), Hangzhou, Zhejiang, China.

Corresponding Author:

Email: xinhuasunqice@163.com (QS)

Email: drck2012@126.com (KC)

[¶]These authors contribute equally to this study.

[*] These authors are corresponding authors.

Unveiling the Wealth-Bone Connection: How Socioeconomic Status Influences Trabecular Bone Health

Abstract

Background

Currently, the relationship between socioeconomic status (SES) and trabecular bone score (TBS) remains unclear. This study uses data from the National Health and Nutrition Examination Survey (NHANES) to explore the connection between SES (poverty income ratio, PIR) and TBS among American adults.

Methods

This study analyzed the relationship between SES and TBS in 7,832 adults using data from the 2005-2008 NHANES. TBS was measured by assessing grayscale variations in lumbar spine dual-energy X-ray absorptiometry (DXA) scans. The study employed a multivariable linear regression model, adjusting for various confounding factors, to examine the association between the PIR and TBS. Additionally, demographic characteristics were described, stratified analyses, single factor analysis, smooth curve fitting, and interaction analysis were conducted.

Results

Using a multivariate linear regression model, after adjusting for confounding factors, the association between SES and TBS was analyzed. Results showed that PIR was positively correlated with TBS (β = 0.0019, 95% CI 0.0002–0.0035, $P < 0.05$), and high-income individuals had a TBS 0.018 higher than low-income individuals (95% CI 0.0106–0.0254, $P < 0.001$). Smooth curve fitting analysis revealed a linear relationship between PIR and TBS, and stratified analysis showed that this positive correlation was particularly evident in Mexican Americans, non-Hispanic whites and non-Hispanic blacks aged 50 and older.

Conclusions

Our research shows that there is a significant positive correlation between adults' SES and TBS. Higher household income levels usually correspond to better TBS levels, which can help prevent osteoporosis and fractures. When developing strategies for the prevention and treatment of osteoporosis, healthcare professionals and policymakers should consider the impact of socioeconomic inequalities.

Keywords

Trabecular bone score; NHANES; Poverty income ratio; Osteoporosis; Socioeconomic status

Introduction

Osteoporosis is a systemic metabolic bone disease characterized by decreased bone density and deterioration of bone microstructure, which often leads to increased bone fragility and a higher risk of fractures [1-2]. A population-based study found that among Americans aged 50 and older, 2.6 million had hip fractures, 14.6 million had wrist fractures, and 5.2 million had vertebral fractures [3]. As the population ages, fracture prevalence continues to rise. Currently, an estimated 14 million Americans and over 200 million people worldwide have osteoporosis [4-5]. Osteoporosis diagnosis traditionally relies on bone mineral density (BMD) measurements, with hip and vertebral fractures being the most severe outcomes. The Trabecular Bone Score (TBS) is a new FDA-approved tool that assesses bone microarchitecture using pixel greyscale analysis of lumbar dual-energy X-ray absorptiometry (DXA) scans [6]. TBS evaluates bone strength and predicts fracture risk partly independently of BMD and clinical risk factors, which can potentially change with pharmacological therapy [7-9]. A high TBS indicates strong, fracture-resistant bone microarchitecture, while a low TBS suggests weaker, more fracture-prone bone.

Socioeconomic status (SES) encompasses income, education, employment, and neighborhood socioeconomic characteristics [10]. Among various measures of poverty, household income relative to the federal poverty line more accurately reflects economic conditions. SES has a significant impact on the incidence of osteoporosis, but this factor is often overlooked [11-13]. A study in Spain found that poverty is a clear risk factor for osteoporotic fractures [14]; a recent cross-sectional study further confirmed the importance of SES in osteoporosis management [15]. Research in Louisiana showed that SES is positively correlated with BMD in the general population [16]. However, a 2009 systematic review found conflicting evidence regarding the association of osteoporotic fractures with income and educational level [17]. This inconsistency may be related to study methodologies or the limitation of using BMD as the sole indicator. Although BMD reflects bone density, it does not assess the microarchitecture of trabecular bone, which is equally important for fracture risk. The TBS can independently assess trabecular bone structure, and may therefore be a more effective indicator for osteoporosis and fracture risk. While many studies have explored the impact of SES on BMD, the effect of SES on TBS remains unclear.

This study aims to addresses the gap in recent research on the relationship between family PIR and TBS, as well as osteoporosis risk in the general population. We hypothesize that higher PIR levels may be positively associated with TBS. This study uses nationally representative sample data to examine the relationship between SES and TBS among adults in the United States. The results of this cross-sectional study will help to elucidate the association between SES and TBS, as well as the risk of osteoporosis, providing new insights for prevention strategies.

Methods

Study Population

The NHANES is a nationally representative survey conducted by the National Center for Health Statistics (NCHS). It used a stratified, multistage probability cluster sampling design to select representative samples from the United States civilian population and assess their health or nutritional status [18]. The detailed data from NHANES are publicly available and have been approved by the Ethics Review Board of the NCHS in the United States; therefore, this study did not require separate ethical approval. The survey utilized the 2005-2008 NHANES dataset, focusing on participants over 20 who completed the family income interview and TBS assessment. Excluding those with incomplete family PIR, TBS, and covariate data, it included 7,832 participants. The specific process is shown in Fig 1.

Figure 1. Participant screening flowchart.

Study Variables

We used the family PIR as a measure of participant financial status, calculated by adjusting household income for annual changes in household size, cost of living, and the consumer price index relative

to federally determined poverty thresholds. We divided the family PIR into three levels: low-income level (PIR≤1.49), middle-income level (1.5≤PIR≤3.51), and high-income level (3.52≤PIR≤ 5).

TBS quantifies the three-dimensional microstructure of trabecular bone by analyzing pixel grayscale gradients and calculating local variations in lumbar spine DXA images [6]. The spine scans were obtained using Hologic QDR-4500A fan-beam densitometers (Hologic, Inc., Bedford, Massachusetts). Subsequently, the total TBS score was estimated in adults aged 20 years and older using TBS software (Med-Imap SA TBS Calculator version 2.1.0.2).

Based on prior research [15] and clinical experience, we included the following covariates that might influence the association between family PIR and total TBS: age, gender, race, body mass index (BMI), alkaline phosphatase, total cholesterol, low-density lipoprotein cholesterol (LDL-C), high-density lipoprotein cholesterol (HDL-C), total protein, serum phosphorus, blood urea nitrogen, serum calcium, serum uric acid, serum cotinine, serum cadmium, serum lead, serum mercury, vitamin D (Vit D), C-reactive protein (CRP）, cardiovascular disease, cancer status, and smoking

status. Details of each variable are available on the NHANES website (http://www.cdc.gov/nchs/nhanes.htm).

Statistical Analysis

Our analyses accounted for the complexity of the NHANES survey design by incorporating sampling weights, primary sampling units, and strata. Descriptive statistics were used to summarize continuous and categorical variables. The weighted mean and standard error (SE) were used for continuous variables, while categorical variables were expressed as weighted percentages. Comparison between groups using weighted chi-square test and weighted linear regression. This study employs a weighted multivariate linear regression model to analyze the relationship between PIR and TBS, categorizing PIR into three levels for trend testing. To control for confounding factors, we established three progressively adjusted models: an unadjusted model, a demographically adjusted model, and a fully adjusted model. Subgroup analyses and interaction tests by age, sex, and race were also performed to assess the stability of the association across different populations. Based on the fully adjusted model, a generalized additive model (GAM) and smooth curve fitting were

applied to examine the nature of the relationship between PIR and TBS. For nonlinear relationships, a two-stage linear regression and recursive algorithm were used to determine the inflection point between PIR and TBS.

A p-value less than 0.05 (two-sided) was considered statistically significant. All statistical analyses were performed using EmpowerStats (http://www.empowerstats.com) and R software (http://www.R-project.org).

Results

Baseline characteristics of study participants

This study included a total of 7,832 people, with 3,847 women and 3,985 men. Table 1 shows the demographic and clinical characteristics weighted by PIR quartiles. Significant differences were observed in the baseline characteristics of the PIR quartiles, except for the serum phosphorus, serum uric acid, BMI, LDL-C, and cardiovascular diseases. Notably, participants in the lower PIR household level were younger, had a higher proportion of women, and were more likely to belong to Non-Hispanic white groups. They had insufficient calcium intake, lower HDL-C and total cholesterol, were more prone to vitamin D deficiency, and were more likely to smoke or be exposed to smoking than those in the high PIR group.

Table 1. Characteristics of the study participants according to PIR levels.

Characteristic	Q1	Q2	Q3	Q4	P value
Age (years)	42.73 ± 16.83	47.73 ± 18.79	45.52 ± 15.99	47.35 ± 13.40	<0.0001
Gender (%)					0.0025
Male	46.81	46.49	49.59	51.62	
Female	53.19	53.51	50.41	48.38	
Race (%)					<0.0001
Mexican American	18.28	11.18	5.51	2.84	
Other race/ethnicity	12.82	11.44	8.87	7.58	
Non-Hispanic white	53.49	63.76	74.25	83.66	
Non-Hispanic black	15.42	13.61	11.37	5.93	
Alkaline Phosphatase (U/L)	72.32 ± 24.65	69.76 ± 23.92	65.86 ± 23.42	65.20 ± 21.28	<0.0001
Blood Urea Nitrogen (mg/dL)	11.80 ± 5.71	12.92 ± 5.32	12.73 ± 4.81	13.12 ± 4.44	<0.0001
Serum Calcium (mg/dL)	9.45 ± 0.37	9.49 ± 0.37	9.47 ± 0.35	9.45 ± 0.35	0.0035
Serum Phosphorus (mg/dL)	3.80 ± 0.58	3.77 ± 0.59	3.79 ± 0.54	3.80 ± 0.56	0.4598
Total Cholesterol (mg/dL)	198.92 ± 45.99	200.77 ± 42.69	198.72 ± 40.64	202.08 ± 38.22	0.019
HDL-C (mg/dL)	50.12 ± 15.64	52.99 ± 16.34	52.71 ± 15.52	54.18 ± 15.78	<0.0001
LDL-C (mg/dL)	118.70 ± 40.69	118.67 ± 37.66	117.25 ± 36.03	118.90 ± 34.08	0.4168
Total Protein (g/L)	7.18 ± 0.47	7.15 ± 0.45	7.14 ± 0.44	7.06 ± 0.43	<0.0001
Serum Uric Acid (mg/dL)	5.37 ± 1.41	5.49 ± 1.42	5.47 ± 1.39	5.43 ± 1.32	0.0926
Serum Cotinine (ng/mL)	108.00 ± 159.75	73.85 ± 142.48	69.02 ± 136.36	46.48 ± 116.11	<0.0001
Serum Cadmium (µg/L)	0.73 ± 0.91	0.59 ± 0.66	0.55 ± 0.69	0.44 ± 0.45	<0.0001
Serum Lead (µg/L)	1.92 ± 1.69	1.83 ± 1.47	1.70 ± 1.41	1.61 ± 1.13	<0.0001
Serum Mercury (µg/L)	1.20 ± 1.73	1.40 ± 1.91	1.54 ± 2.02	2.22 ± 2.56	<0.0001
CRP (mg/L)	0.45 ± 0.85	0.40 ± 0.89	0.36 ± 0.62	0.36 ± 0.83	0.0016
BMI (Kg/m2)	28.11 ± 6.38	28.24 ± 5.79	28.18 ± 5.92	27.96 ± 5.46	0.373
Vit D (nmol/L)	58.71 ± 23.17	61.31 ± 23.74	64.31 ± 23.64	67.56 ± 22.72	<0.0001
TBS	1.37 ± 0.15	1.37 ± 0.15	1.39 ± 0.14	1.39 ± 0.14	<0.0001

Cardiovascular Diseases(%)					0.6385
Yes	2.97	3.33	2.68	2.74	
No	97.03	96.67	97.32	97.26	
Cancer(%)					0.0483
Yes	7.29	8.37	7.61	9.47	
No	92.71	91.63	92.39	90.53	
Smoking status					<0.0001
Every day	33.75	23.01	20.98	12.2	
Some days	4.92	3.45	3.46	3.19	
Not at all	17.05	26.01	23.3	28.22	
Missing	44.28	47.54	52.25	56.39	

Data are expressed as weighted means ± SE or percentages (%).

Abbreviation: SE, standard error; HDL-C, high-density lipoprotein cholesterol; LDL-C, low-density lipoprotein cholesterol; CRP, C-reactive protein; Vit D, vitamin D; BMI, body mass index; TBS, trabecular bone score.

Associations between family PIR and total TBS

Table 2 shows the regression analysis results of family PIR and TBS. In the unadjusted model, PIR was significantly positively correlated with TBS (β = 0.0046, 95% CI: 0.0026-0.0066, $P < 0.001$). After adjusting for covariates, models 2 and 3 still maintained this significant correlation. In the fully adjusted model (model 3), for each unit increase in PIR, TBS increased by 0.0019 (95% CI: -0.0273-0.0222, $P<0.05$). When grouped by income level, high-income families had a TBS higher by 0.018 compared to low-income families. Meanwhile, the trend test showed a P-value of $P < 0.001$, indicating that TBS significantly increases as family PIR levels rise. Moreover, in the generalized additive model and smoothing curve fitting, we found a linear relationship between PIR and TBS (Fig 2).

Fig 2. The association between family PIR and total TBS. (A) Each black point represents a sample. (B) The solid red line represents the smooth curve fit between variables. Blue bands represent the 95% of confidence interval from the fit. The model adjusts all variables except family PIR and total TBS.

Stratified analyses based on additional variables

Table 3 and Fig 3 shows the results of stratified analysis by age, gender, and race. In the unadjusted model, all age groups showed a significant positive correlation between household PIR and TBS. However, after full adjustment, only the≥50 years group maintained significance. Regarding gender, both males and females showed significant positive correlations after full adjustment. In the racial analysis, only non-Hispanic whites showed a significant association in the unadjusted model, while Mexican Americans, non-Hispanic whites, and non-Hispanic blacks all showed significant associations after full adjustment. Other racial groups showed no statistical significance before or after adjustment.

Figure 3. The association between family PIR and total TBS stratified by age (A), gender (B) and race/ethnicity (C). This model adjusted for all variables except family PIR, total TBS, and corresponding stratified variables.

Table 2. Association between the PIR and the TBS.

Variable	Model 1 β (95% CI)	Model 2 β (95% CI)	Model 3 β (95% CI)
Family PIR per 1 increase	0.0046 (0.0026, 0.0066)***	0.0064 (0.0046, 0.0082)***	0.0019 (0.0002, 0.0035)*
Low-income level (0 - 1.49)	Ref	Ref	Ref
Middle-income level (1.5 - 3.51)	-0.0058 (-0.0165, 0.0049)	0.0140 (0.0045, 0.0236)**	0.0099 (0.0021, 0.0176)*
High-income level (3.52 - 5)	0.0145 (0.0044, 0.0246)**	0.0238 (0.0147, 0.0328)***	0.0180 (0.0106, 0.0254)***
P for trend	<0.001	<0.001	<0.001

Model 1: unadjusted;

Model 2: adjusted for age, gender, and race;

Model 3: further adjusted for alkaline phosphatase, blood urea nitrogen, serum calcium, total cholesterol, serum phosphorus, serum uric acid, total protein, serum cotinine, HDL-C, LDL-C, BMI, VIT D, CRP, serum cadmium, serum lead, serum mercury, cardiovascular diseases, cancer, smoking status. $P < 0.05$ presents significant difference, with *p < 0.05, **p < 0.01, ***p < 0.001.

.

Table 3. Association of family PIR and TBS after stratification by age, gender, and race.

Subgroup	N	Model 1 β (95% CI)	Model 2 β (95% CI)	Model 3 β (95% CI)
Age	7832			
>=20, <35	1923	0.0065 (0.0034, 0.0096)***	0.0043 (0.0012, 0.0075)**	0.0024 (-0.0004, 0.0051)
>=35, <50	2145	0.0089 (0.0054, 0.0124)***	0.0091 (0.0055, 0.0127)***	0.0018 (-0.0012, 0.0048)
>=50	3764	0.0123 (0.0095, 0.0152)***	0.0121 (0.0092, 0.0151)***	0.0104 (0.0078, 0.0129)***
Total	7832	0.0096 (0.0078, 0.0114)***	0.0091 (0.0072, 0.0109)***	0.0055 (0.0039, 0.0071)***
Gender				
Male	3985	-0.0002 (-0.0031, 0.0026)	0.0033 (0.0006, 0.0060)*	0.0050 (0.0028, 0.0071)***
Female	3847	0.0095 (0.0067, 0.0123)***	0.0091 (0.0066, 0.0116)***	0.0038 (0.0017, 0.0060)***
Total	7832	0.0047 (0.0027, 0.0068)***	0.0064 (0.0046, 0.0082)***	0.0037 (0.0021, 0.0052)***
Race				
Mexican American	1426	0.0019 (-0.0031, 0.0069)	0.0060 (0.0015, 0.0106)**	0.0073 (0.0035, 0.0111)***
Other race/ethnicity	884	-0.0009 (-0.0064, 0.0045)	0.0033 (-0.0016, 0.0083)	0.0003 (-0.0040, 0.0045)
Non-Hispanic white	3862	0.0069 (0.0039, 0.0099)***	0.0073 (0.0047, 0.0099)***	0.0039 (0.0017, 0.0061)***
Non-Hispanic black	1660	0.0013 (-0.0033, 0.0060)	0.0031 (-0.0012, 0.0073)	0.0041 (0.0005, 0.0076)*
Total	7832	0.0052 (0.0031, 0.0072)***	0.0064 (0.0046, 0.0082)***	0.0037 (0.0021, 0.0052)***

Model 1: unadjusted.

Model 2: adjusted for age, gender, and race.

Model 3: adjusted for all variables except family PIR, total TBS, and corresponding stratified variables. $P < 0.05$ presents significant difference, with *p < 0.05, **p < 0.01, ***p < 0.001.

Discussion

In our study, we investigated the association between family PIR and total TBS among a large sample of the U.S. population over the age of 20. Our results showed that family PIR, whether as a continuous or categorical variable, was positively and linearly associated with total TBS when adjusted for potential confounding factors. The lower family income subgroup was younger, more often female, and more likely to belong to Non-Hispanic whites. They were also more likely to be daily smokers, suffer from vitamin D deficiency, and be exposed to environmental pollution more frequently than those in the higher family PIR category. After full adjustment, a significant association between family PIR and total TBS was found among participants aged ≥ 50, including both men and women, as well as Mexican Americans, non-Hispanic whites, and non-Hispanic blacks. This suggests that a higher family PIR is associated with a lower probability of osteoporosis and osteoporotic fractures.

Although the effect size observed in this study was small, its clinical significance remains notable. As a continuous scale measurement, the range of TBS is relatively narrow, so even minor differences may influence the diagnosis of bone microarchitecture deterioration.

Notably, this effect reflects the independent contribution of SES after adjusting for multiple factors, suggesting that the overall impact of SES on bone health may be more profound. From a population health perspective, subtle shifts in the distribution of risk factors can lead to substantial changes in disease burden. Given that SES affects a broad population, even small effects may accumulate to yield considerable public health implications for fracture prevention. The consistent associations observed across multiple subgroups in this study further affirm the clinical relevance of these findings.

Osteoporosis risk is partially determined by the cumulative effects of unhealthy dietary and activity behaviors occurring throughout life. TBS is a new complementary approach for osteoporosis evaluation in clinical practice. Several mechanisms may explain the relationship of family SES to bone health. First of all, it is likely that adults of low socioeconomic position may be at increased osteoporosis risk because of inadequate nutrition, calcium intake, and physical activity during childhood, resulting in suboptimal achievement of peak bone mass earlier in life [19-20]. Lower socioeconomic status, poverty, food insecurity, inadequate nutrition, and calcium plus vitamin D deficiency in older adulthood may result in accelerated loss of bone mass and accelerated development of

osteoporosis with age [21-22]. Evidence from a cohort study involving 246 women with osteoporosis demonstrated that 25-hydroxyvitamin D (25OHD) was associated with spine TBS, affecting bone microarchitecture and mobility performance [23]. Secondly, higher income was associated with higher self-reported physical activity [24]. Economically active individuals may engage in regular exercise, which helps improve balance and postural stability, potentially resulting in a lower risk of osteoporosis [25-26]. A cohort study from Australia involving 894 men and 682 women (ages 24-98 years) showed that lower TBS was associated with older age, increased weight, lower childhood physical activity, and lower BMD in men, and with older age, shorter stature, lower BMD, and lower mobility in women [27].

Smoking has long been identified as a risk factor for osteoporosis [28]. A cross-sectional study including 10,564 participants showed a nonlinear and positive relationship between higher serum cotinine levels (≥3 ng/ml) and the prevalence of osteoporosis [29]. Additionally, individuals in lower income jobs are more likely to carry a higher risk of occupational injury and may have workplace exposure to environmental risk factors, including hazardous wastes and toxic chemical pollution (i.e., lead), as they are more

likely to have industrial jobs [30]. This study found that serum cadmium and lead levels were significantly elevated in the low PIR group (Table 1). Heavy metal exposure may inhibit bone formation by downregulating runt-related transcription factor 2 (RUNX2) expression [31] and activate the nuclear factor kappa-B (NF-κB) pathway to promote bone resorption [32-33], thereby exacerbating the deterioration of bone microstructure. A large cross-sectional study revealed a dose-dependent relationship between TBS and bone cadmium (B-Cd) for both males and females [34].

Moreover, working individuals, citizens, and those with higher economic status were more likely to have better access to healthcare through health insurance. High-income individuals can afford the treatment and management costs of chronic diseases such as diabetes, hypertension, and osteoporosis, and take timely preventive measures and treatment [35]. In contrast, persons who lack insurance receive less medical care, including screening and treatment, and may receive poorer-quality care [36]. Compared to those with less education, well-educated respondents are more likely to be employed, earn higher incomes, and experience less economic hardship. They also tend to have extensive health knowledge, contributing to a healthier lifestyle [37]. Overall,

citizenship, working status, and economic status may serve as surrogate markers for the healthcare axis and a better lifestyle, revealing the relationship between family SES and total TBS from multiple perspectives. In summary, individuals with lower family SES may face challenges in areas such as nutritional intake, lifestyle, work-life environment, chronic disease management, and health beliefs, which can lead to osteoporosis. To clarify the relationship between family SES and total TBS, it is necessary to conduct more empirical research to understand how these factors interact and influence bone health.

Our study still has several limitations. First of all, due to its cross-sectional nature, we were unable to determine the temporal relationship between PIR and TBS. Secondly, the included covariates may not be completely accurate, which can affect the accuracy of the results. Despite these shortcomings, there are some strengths in our study, such as the fact that it is founded on a great deal of representative data from NHANES. We have demonstrated a positive association between family PIR and total TBS status. Additionally, we were able to undertake subgroup analyses of family PIR and total TBS across age, gender, and ethnicity, which has diagnostic value in predicting the presence of osteoporosis in a

patient.

Conclusion

Our results suggest that family PIR positively correlated with total TBS among U.S adults. Differences of PIR in the population should be considered in the diagnosis and treatment of osteoporosis.

Author statements

Acknowledgements

The author thanks the NHANES staff and participants for their valuable contributions.

Ethical approval

The NHANES data is publicly accessible. Per local regulations, this study was exempt from ethical review and approval.

Data availability statement

This study utilized the publicly available dataset from the NHANES in the United States (http://www.cdc.gov/nchs/nhanes/). The data were anonymized prior to analysis, participants provided informed consent, and approval was obtained from the Institutional Review Board of the National Center for Health Statistics.

Author contributions

Conceptualization: Yuan Zhang, Qice Sun, and Yanlei Li.

Data curation: Yanlei Li and Kai Chen.

Funding acquisition: Yuan Zhang and Qice Sun.

Investigation: Yuan Zhang, Tingxiao Zhao, and Qice Sun.

Methodology: Yanlei Li and Kai Chen.

Project administration: Yuan Zhang and Qice Sun.

Resources: Yuan Zhang, Tingxiao Zhao, and Qice Sun.

Software: Yanlei Li, Tingxiao Zhao, and Qice Sun.

Supervision: Yuan Zhang and Qice Sun.

Validation: Yuan Zhang, Tingxiao Zhao, and Qice Sun.

Writing-original draft: Yuan Zhang, Tingxiao Zhao, and Qice Sun.

Writing-review & editing: Yuan Zhang and Qice Sun.

References

[1] Consensus development conference: diagnosis, prophylaxis, and treatment of osteoporosis. Am J Med. 1993;94:646-650. doi:10.1016/0002-9343(93)90218-e.

[2] Kanis JA, McCloskey EV, Harvey NC, Johansson H, Leslie WD. Intervention Thresholds and the Diagnosis of Osteoporosis. J Bone Miner Res. 2015;30:1747-1753. doi:10.1002/jbmr.2531.

[3] Xu B, Radojčić MR, Anderson DB, Shi B, Yao L, Chen Y et al. Trends in prevalence of fractures among adults in the United States, 1999-2020: a population-based study. Int J Surg. 2024;110:721-732. doi: 10.1097/JS9.0000000000000883.

[4] Shen Y, Huang X, Wu J, Lin X, Zhou X, Zhu Z, et al. The Global Burden of Osteoporosis, Low Bone Mass, and Its Related Fracture in 204 Countries and Territories, 1990-2019. Front Endocrinol (Lausanne). 2022; 13:882241. doi:10.3389/fendo.2022.882241.

[5] Rosen CJ. The Epidemiology and Pathogenesis of Osteoporosis. In: Feingold KR, Ahmed SF, Anawalt B, et al., eds. Endotext. South Dartmouth (MA): MDText.com, Inc.; June 21, 2020.

[6] Pothuaud L, Carceller P, Hans D. Correlations between grey-level variations in 2D projection images (TBS) and 3D microarchitecture: applications in the study of human trabecular bone microarchitecture. Bone. 2008;42:775-787. doi:10.1016/j.bone.2007.11.018.

[7] Shevroja E, Reginster JY, Lamy O, Al-Daghri N, Chandran M, Demoux-Baiada AL, et al. Update on the clinical use of trabecular bone score (TBS) in the management of osteoporosis: results of an expert group meeting organized by the European Society for

Clinical and Economic Aspects of Osteoporosis, Osteoarthritis and Musculoskeletal Diseases (ESCEO), and the International Osteoporosis Foundation (IOF) under the auspices of WHO Collaborating Center for Epidemiology of Musculoskeletal Health and Aging. Osteoporos Int. 2023;34:1501-1529. doi:10.1007/s00198-023-06817-4.

[8] Leslie WD, Shevroja E, Johansson H, McCloskey EV, Harvey NC, Kanis JA, et al. Risk-equivalent T-score adjustment for using lumbar spine trabecular bone score (TBS): the Manitoba BMD registry. Osteoporos Int. 2018;29:751-758. doi:10.1007/s00198-018-4405-0.

[9] Shevroja E, Lamy O, Kohlmeier L, Koromani F, Rivadeneira F, Hans D. Use of Trabecular Bone Score (TBS) as a Complementary Approach to Dual-energy X-ray Absorptiometry (DXA) for Fracture Risk Assessment in Clinical Practice. J Clin Densitom. 2017;20:334-345. doi:10.1016/j.jocd.2017.06.019.

[10] Adler NE, Newman K. Socioeconomic disparities in health: pathways and policies. Health Aff (Millwood). 2002;21:60-76. doi:10.1377/hlthaff.21.2.60.

[11] Du Y, Zhao LJ, Xu Q, Wu KH, Deng HW. Socioeconomic status and bone mineral density in adults by race/ethnicity and gender: the Louisiana osteoporosis study. Osteoporos Int. 2017;28:1699-1709. doi:10.1007/s00198-017-3951-1.

[12] Brennan SL, Pasco JA, Urquhart DM, Oldenburg B, Wang Y, Wluka AE. Association between socioeconomic status and bone mineral density in adults: a systematic review. Osteoporos Int. 2011;22:517-527. doi:10.1007/s00198-010-1261-y.

[13] Xiao PL, Fuerwa C, Hsu CJ, Peng R, Cui AY, Jiang N, et al. Socioeconomic status influences on bone mineral density in American men: findings from NHANES 2011-2020.

Osteoporos Int. 2022;33:2347-2355. doi:10.1007/s00198-022-06498-5.

[13] Adler NE, Newman K. Socioeconomic disparities in health: pathways and policies. Health Aff (Millwood). 2002;21:60-76. doi:10.1377/hlthaff.21.2.60.

[14] Navarro MC, Sosa M, Saavedra P, Lainez P, Marrero M, Torres M, et al. Poverty is a risk factor for osteoporotic fractures. Osteoporos Int. 2009;20:393–398. doi: 10.1007/s00198-008-0697-9.

[15] Xiao PL, Fuerwa C, Hsu CJ, Peng R, Cui AY, Jiang N, et al. Socioeconomic status influences on bone mineral density in American men: findings from NHANES 2011-2020. Osteoporos Int. 2022;33:2347-2355. doi: 10.1007/s00198-022-06498-5.

[16] Du Y, Zhao LJ, Xu Q, Wu KH, Deng HW. Socioeconomic status and bone mineral density in adults by race/ethnicity and gender: the Louisiana osteoporosis study. Osteoporos Int. 2017;28:1699-1709. doi: 10.1007/s00198-017-3951-1.

[17] Brennan SL, Pasco JA, Urquhart DM, Oldenburg B, Hanna F, Wluka AE. The association between socioeconomic status and osteoporotic fracture in population-based adults: a systematic review. Osteoporos Int. 2009;20:1487-97. doi: 10.1007/s00198-008-0822-9.

[18] NHANES-National Health and Nutrition Examination Survey Homepage. https://www.cdc.gov/nchs/nhanes/index.htm. Published November 28, 2023. Accessed January 3, 2024.

[19] Weaver CM, Gordon CM, Janz KF, Kalkwarf HJ, Lappe JM, Lewis R, et al. The National Osteoporosis Foundation’s position statement on peak bone mass development and lifestyle factors: a systematic review and implementation recommendations. Osteoporos

Int. 2016;27:1281-1386. doi:10.1007/s00198-015-3440-3.

[20] Viljakainen HT. Factors influencing bone mass accrual: focus on nutritional aspects. Proc Nutr Soc. 2016;75:415-419. doi:10.1017/S0029665116000252.

[21] Muñoz-Garach A, García-Fontana B, Muñoz-Torres M. Nutrients and Dietary Patterns Related to Osteoporosis. Nutrients. 2020;12:1986. doi:10.3390/nu12071986.

[22] Weaver CM, Alexander DD, Boushey CJ, Dawson-Hughes B, Lappe JM, LeBoff MS, et al. Calcium plus vitamin D supplementation and risk of fractures: an updated meta-analysis from the National Osteoporosis Foundation. Osteoporos Int. 2016;27:367-376. doi:10.1007/s00198-015-3386-5.

[23] Haeri NS, Perera S, Greenspan SL. The association of vitamin D with bone microarchitecture, muscle strength, and mobility performance in older women in long-term care. Bone. 2023;176:116867. doi:10.1016/j.bone.2023.116867.

[24] Kari JT, Pehkonen J, Hirvensalo M, Yang X, Hutri-Kähönen N, Raitakari OT, et al. Income and Physical Activity among Adults: Evidence from Self-Reported and Pedometer-Based Physical Activity Measurements. Harezlak J, ed. PLoS ONE. 2015;10:e0135651. doi:10.1371/journal.pone.0135651.

[25] Kai MC, Anderson M, Lau EM. Exercise interventions: defusing the world's osteoporosis time bomb. Bull World Health Organ. 2003;81(11):827-830.

[26] Pinheiro MB, Oliveira J, Bauman A, Fairhall N, Kwok W, Sherrington C. Evidence on physical activity and osteoporosis prevention for people aged 65+ years: a systematic review to inform the WHO guidelines on physical activity and sedentary behaviour. Int J Behav Nutr Phys Act. 2020;17:150. doi:10.1186/s12966-020-01040-4.

[27] Anderson KB, Holloway-Kew KL, Hans D, Kotowicz MA, Hyde NK, Pasco JA. Physical and lifestyle factors associated with trabecular bone score values. Arch Osteoporos. 2020;15:177. doi:10.1007/s11657-020-00841-1.

[28] Cusano NE. Skeletal Effects of Smoking. Curr Osteoporos Rep. 2015;13:302-309. doi:10.1007/s11914-015-0278-8.

[29] Hou W, Chen S, Zhu C, Gu Y, Zhu L, Zhou Z. Associations between smoke exposure and osteoporosis or osteopenia in a US NHANES population of elderly individuals. Front Endocrinol (Lausanne). 2023;14:1074574. doi:10.3389/fendo.2023.1074574.

[30] Fuller R, Landrigan PJ, Balakrishnan K, Bathan G, Bose-O'Reilly S, Brauer M, et al. Pollution and health: a progress update. Lancet Planet Health. 2022;6:e535-e547. doi:10.1016/S2542-5196(22)00090-0.

[31] Jalili C, Kazemi M, Taheri E, Mohammadi H, Boozari B, Hadi A, et al. Exposure to heavy metals and the risk of osteopenia or osteoporosis: a systematic review and meta-analysis. Osteoporos Int. 2020;31:1671-1682. doi:10.1007/s00198-020-05429-6.

[32] Luo H, Gu R, Ouyang H, Wang L, Shi S, Ji Y, et al. Cadmium exposure induces osteoporosis through cellular senescence, associated with activation of NF-κB pathway and mitochondrial dysfunction. Environ Pollut. 2021;290:118043. doi: 10.1016/j.envpol.2021.

[33] Scimeca M, Feola M, Romano L, Rao C, Gasbarra E, Bonanno E, et al. Heavy metals accumulation affects bone microarchitecture in osteoporotic patients. Environ Toxicol. 2017;32:1333-1342. doi:10.1002/tox.22327.

[34] Huang JW, Fang WH, Chen WL. Detrimental Association Between Blood Cadmium

Concentration and Trabecular Bone Score. Biol Trace Elem Res. 2023;201:82-89. doi:10.1007/s12011-022-03143-4.

[35] Curtis LJ. An economic perspective on the causal explanations for the socioeconomic inequalities in health. Rev Panam Salud Publica. 2018;42:e53. doi:10.26633/RPSP.2018.53.

[36] Burstin HR, Lipsitz SR, Brennan TA. Socioeconomic status and risk for substandard medical care. JAMA. 1992;268:2383-2387.

[37] Zajacova A, Lawrence EM. The relationship between education and health: reducing disparities through a contextual approach. Annual review of public health. 2018;39:273. doi:10.1146/annurev-publhealth-031816-044628.

Figure 1

Click here to access/download;Figure;Fig1..tiff

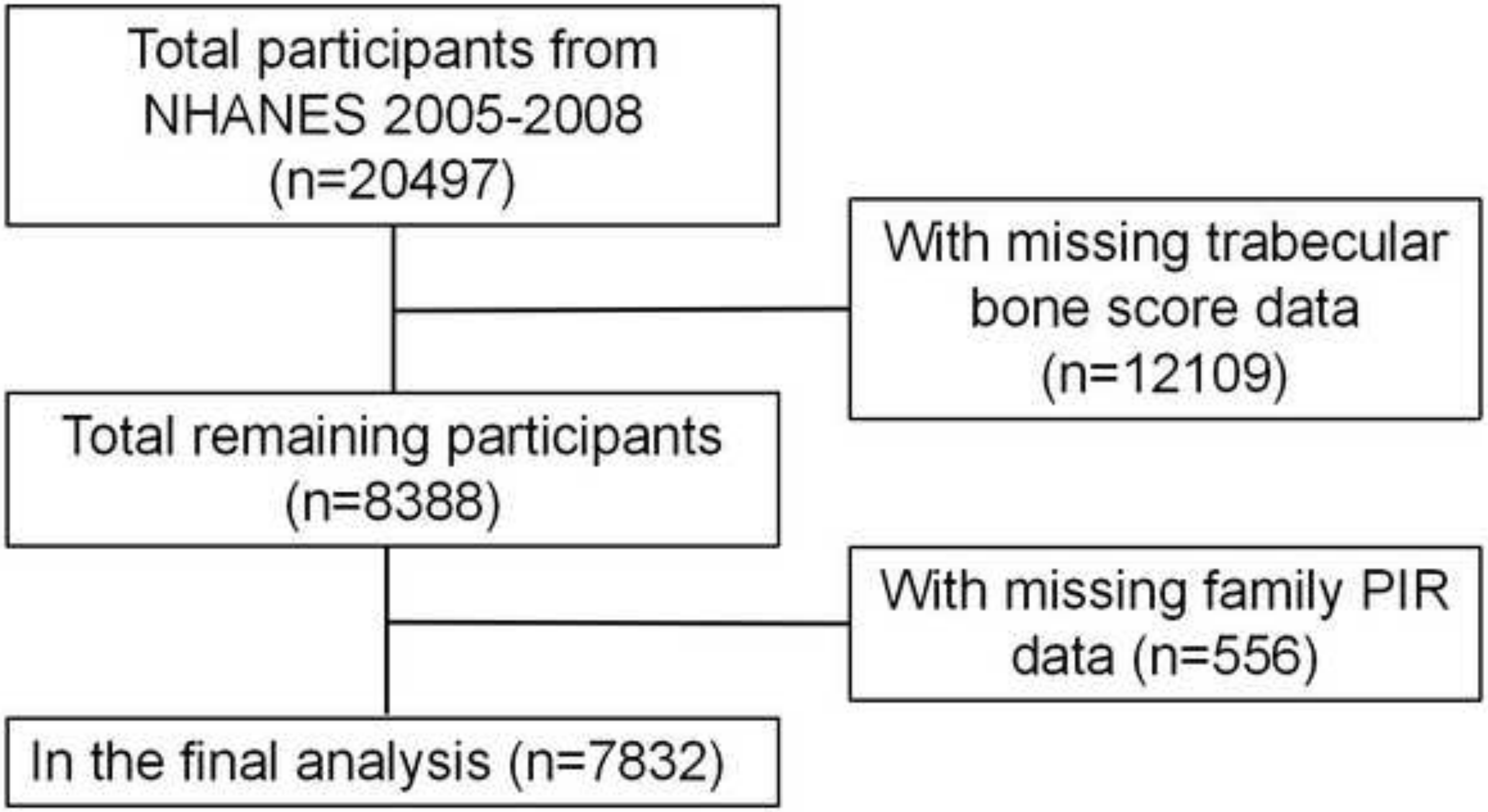

Figure 2

Click here to access/download;Figure;Fig 2.tiff

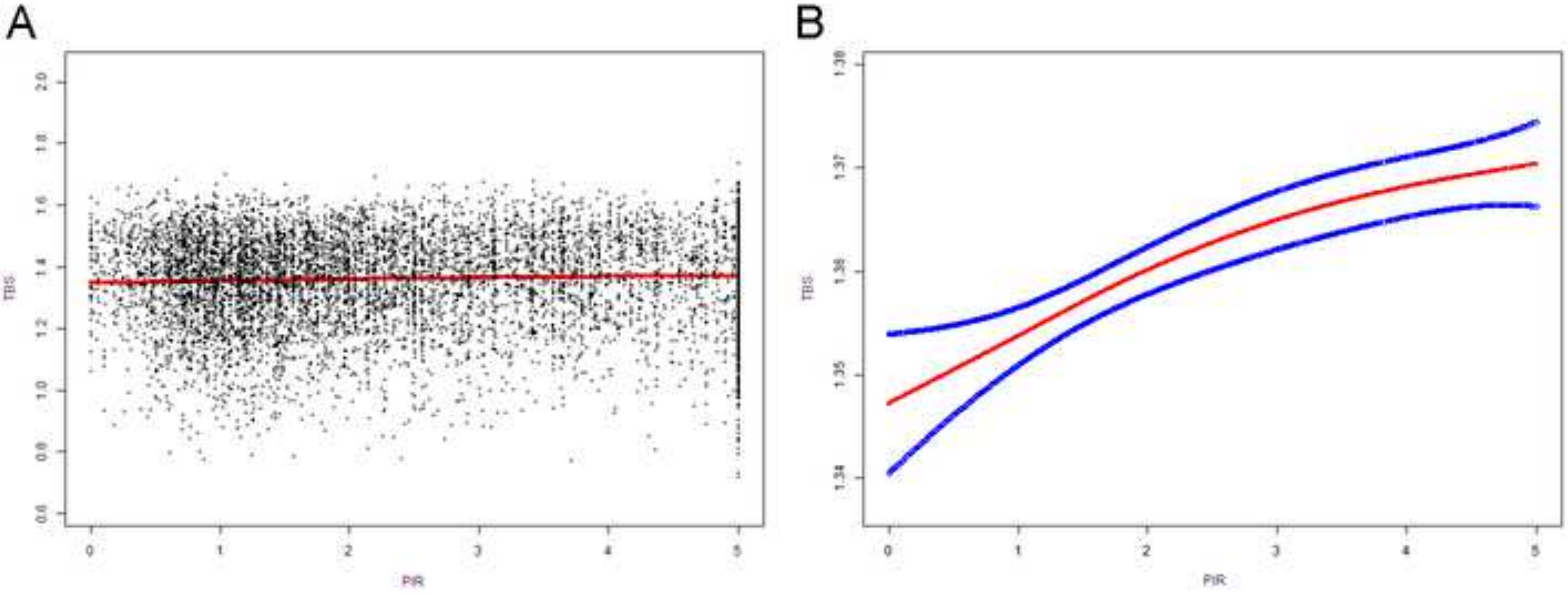

Figure 3

Click here to access/download;Figure;Fig 3..tiff

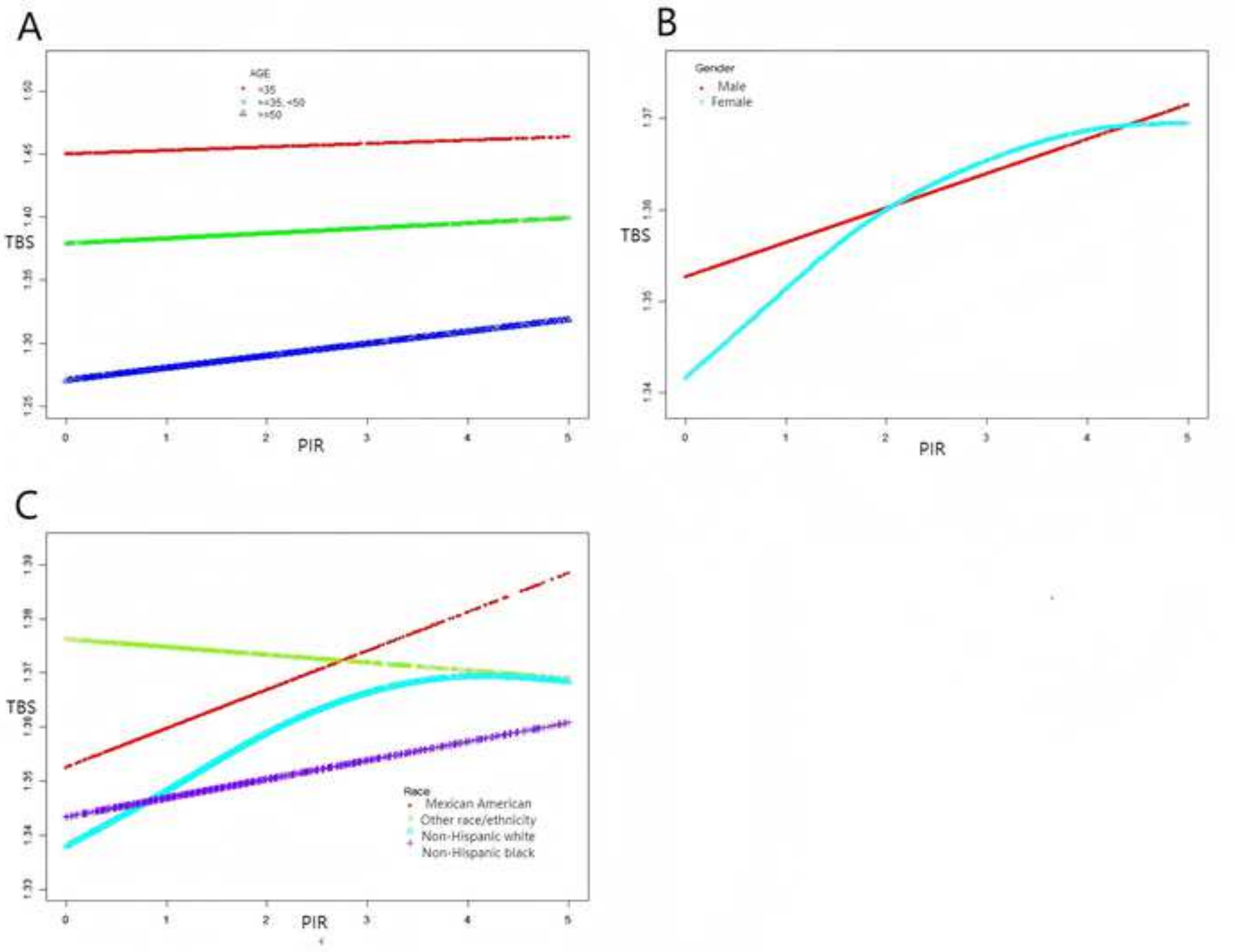

Title of Manuscript:

Unveiling the Wealth-Bone Connection: How Socioeconomic Status Influences Trabecular Bone Health

Author List:

Yuan Zhang[1¶], Tingxiao Zhao[2¶], Yanlei Li[2], Kai Chen[1*], Qice Sun[1*].

Affiliations:

1. Department of Rheumatology, The Second Affiliated Hospital of Zhejiang Chinese Medical University, Hangzhou, China.

2. Center for Plastic & Reconstructive Surgery, Department of Orthopedics, Zhejiang Provincial People's Hospital (Affiliated People's Hospital, Hangzhou Medical College), Hangzhou, Zhejiang, China.

Corresponding Author:

Email: xinhuasunqice@163.com (QS)

Email: drck2012@126.com (KC)

[¶]These authors contribute equally to this study.

[*] These authors are corresponding authors.

Unveiling the Wealth-Bone Connection: How Socioeconomic Status Influences Trabecular Bone Health

Abstract

Background

Currently, the relationship between socioeconomic status (SES) and trabecular bone score (TBS) remains unclear. This study uses data from the National Health and Nutrition Examination Survey (NHANES) to explore the connection between SES (poverty income ratio, PIR) and TBS among American adults.

Methods

This study analyzed the relationship between SES and TBS in 7,832 adults using data from the 2005-2008 NHANES. TBS was measured by assessing grayscale variations in lumbar spine dual-energy X-ray absorptiometry (DXA) scans. The study employed a multivariable linear regression model, adjusting for various confounding factors, to examine the association between the PIR and TBS. Additionally, demographic characteristics were described, stratified analyses, single factor analysis, smooth curve fitting, and interaction analysis were conducted.

Results

Using a multivariate linear regression model, after adjusting for confounding factors, the association between SES and TBS was analyzed. Results showed that PIR was positively correlated with TBS (β = 0.0019, 95% CI 0.0002–0.0035, $P < 0.05$), and high-income individuals had a TBS 0.018 higher than low-income individuals (95% CI 0.0106–0.0254, $P < 0.001$). Smooth curve fitting analysis revealed a linear relationship between PIR and TBS, and stratified analysis showed that this positive correlation was particularly evident in Mexican Americans, non-Hispanic whites and non-Hispanic blacks aged 50 and older.

Conclusions

Our research shows that there is a significant positive correlation between adults' SES and TBS. Higher household income levels usually correspond to better TBS levels, which can help prevent osteoporosis and fractures. When developing strategies for the prevention and treatment of osteoporosis, healthcare professionals and policymakers should consider the impact of socioeconomic inequalities.

Keywords

Trabecular bone score; NHANES; Poverty income ratio; Osteoporosis; Socioeconomic status

Introduction

Osteoporosis is a systemic metabolic bone disease characterized by decreased bone density and deterioration of bone microstructure, which often leads to increased bone fragility and a higher risk of fractures [1-2]. A population-based study found that among Americans aged 50 and older, 2.6 million had hip fractures, 14.6 million had wrist fractures, and 5.2 million had vertebral fractures [3]. As the population ages, fracture prevalence continues to rise. Currently, an estimated 14 million Americans and over 200 million people worldwide have osteoporosis [4-5]. Osteoporosis diagnosis traditionally relies on bone mineral density (BMD) measurements, with hip and vertebral fractures being the most severe outcomes. The Trabecular Bone Score (TBS) is a new FDA-approved tool that assesses bone microarchitecture using pixel greyscale analysis of lumbar dual-energy X-ray absorptiometry (DXA) scans [6]. TBS evaluates bone strength and predicts fracture risk partly independently of BMD and clinical risk factors, which can potentially change with pharmacological therapy [7-9]. A high TBS indicates strong, fracture-resistant bone microarchitecture, while a low TBS suggests weaker, more fracture-prone bone.

Socioeconomic status (SES) encompasses income, education, employment, and neighborhood socioeconomic characteristics [10]. Among various measures of poverty, household income relative to the federal poverty line more accurately reflects economic conditions. SES has a significant impact on the incidence of osteoporosis, but this factor is often overlooked [11-13]. A study in Spain found that poverty is a clear risk factor for osteoporotic fractures [14]; a recent cross-sectional study further confirmed the importance of SES in osteoporosis management [15]. Research in Louisiana showed that SES is positively correlated with BMD in the general population [16]. However, a 2009 systematic review found conflicting evidence regarding the association of osteoporotic fractures with income and educational level [17]. This inconsistency may be related to study methodologies or the limitation of using BMD as the sole indicator. Although BMD reflects bone density, it does not assess the microarchitecture of trabecular bone, which is equally important for fracture risk. The TBS can independently assess trabecular bone structure, and may therefore be a more effective indicator for osteoporosis and fracture risk. While many studies have explored the impact of SES on BMD, the effect of SES on TBS remains unclear.

This study aims to addresses the gap in recent research on the relationship between family PIR and TBS, as well as osteoporosis risk in the general population. We hypothesize that higher PIR levels may be positively associated with TBS. This study uses nationally representative sample data to examine the relationship between SES and TBS among adults in the United States. The results of this cross-sectional study will help to elucidate the association between SES and TBS, as well as the risk of osteoporosis, providing new insights for prevention strategies.

Methods

Study Population

The NHANES is a nationally representative survey conducted by the National Center for Health Statistics (NCHS). It used a stratified, multistage probability cluster sampling design to select representative samples from the United States civilian population and assess their health or nutritional status [18]. The detailed data from NHANES are publicly available and have been approved by the Ethics Review Board of the NCHS in the United States; therefore, this study did not require separate ethical approval. The survey utilized the 2005-2008 NHANES dataset, focusing on participants over 20 who completed the family income interview and TBS assessment. Excluding those with incomplete family PIR, TBS, and covariate data, it included 7,832 participants. The specific process is shown in Fig 1.

Figure 1. Participant screening flowchart.

Study Variables

We used the family PIR as a measure of participant financial status, calculated by adjusting household income for annual changes in household size, cost of living, and the consumer price index relative

to federally determined poverty thresholds. We divided the family PIR into three levels: low-income level (PIR≤1.49), middle-income level (1.5≤PIR≤3.51), and high-income level (3.52≤PIR≤ 5).

TBS quantifies the three-dimensional microstructure of trabecular bone by analyzing pixel grayscale gradients and calculating local variations in lumbar spine DXA images [6]. The spine scans were obtained using Hologic QDR-4500A fan-beam densitometers (Hologic, Inc., Bedford, Massachusetts). Subsequently, the total TBS score was estimated in adults aged 20 years and older using TBS software (Med-Imap SA TBS Calculator version 2.1.0.2).

Based on prior research [15] and clinical experience, we included the following covariates that might influence the association between family PIR and total TBS: age, gender, race, body mass index (BMI), alkaline phosphatase, total cholesterol, low-density lipoprotein cholesterol (LDL-C), high-density lipoprotein cholesterol (HDL-C), total protein, serum phosphorus, blood urea nitrogen, serum calcium, serum uric acid, serum cotinine, serum cadmium, serum lead, serum mercury, vitamin D (Vit D), C-reactive protein (CRP）, cardiovascular disease, cancer status, and smoking

status. Details of each variable are available on the NHANES website (http://www.cdc.gov/nchs/nhanes.htm).

Statistical Analysis

Our analyses accounted for the complexity of the NHANES survey design by incorporating sampling weights, primary sampling units, and strata. Descriptive statistics were used to summarize continuous and categorical variables. The weighted mean and standard error (SE) were used for continuous variables, while categorical variables were expressed as weighted percentages. Comparison between groups using weighted chi-square test and weighted linear regression. This study employs a weighted multivariate linear regression model to analyze the relationship between PIR and TBS, categorizing PIR into three levels for trend testing. To control for confounding factors, we established three progressively adjusted models: an unadjusted model, a demographically adjusted model, and a fully adjusted model. Subgroup analyses and interaction tests by age, sex, and race were also performed to assess the stability of the association across different populations. Based on the fully adjusted model, a generalized additive model (GAM) and smooth curve fitting were

applied to examine the nature of the relationship between PIR and TBS. For nonlinear relationships, a two-stage linear regression and recursive algorithm were used to determine the inflection point between PIR and TBS.

A p-value less than 0.05 (two-sided) was considered statistically significant. All statistical analyses were performed using EmpowerStats (http://www.empowerstats.com) and R software (http://www.R-project.org).

Results

Baseline characteristics of study participants

This study included a total of 7,832 people, with 3,847 women and 3,985 men. Table 1 shows the demographic and clinical characteristics weighted by PIR quartiles. Significant differences were observed in the baseline characteristics of the PIR quartiles, except for the serum phosphorus, serum uric acid, BMI, LDL-C, and cardiovascular diseases. Notably, participants in the lower PIR household level were younger, had a higher proportion of women, and were more likely to belong to Non-Hispanic white groups. They had insufficient calcium intake, lower HDL-C and total cholesterol, were more prone to vitamin D deficiency, and were more likely to smoke or be exposed to smoking than those in the high PIR group.

Table 1. Characteristics of the study participants according to PIR levels.

Characteristic	Q1	Q2	Q3	Q4	P value
Age (years)	42.73 ± 16.83	47.73 ± 18.79	45.52 ± 15.99	47.35 ± 13.40	<0.0001
Gender (%)					0.0025
Male	46.81	46.49	49.59	51.62	
Female	53.19	53.51	50.41	48.38	
Race (%)					<0.0001
Mexican American	18.28	11.18	5.51	2.84	
Other race/ethnicity	12.82	11.44	8.87	7.58	
Non-Hispanic white	53.49	63.76	74.25	83.66	
Non-Hispanic black	15.42	13.61	11.37	5.93	
Alkaline Phosphatase (U/L)	72.32 ± 24.65	69.76 ± 23.92	65.86 ± 23.42	65.20 ± 21.28	<0.0001
Blood Urea Nitrogen (mg/dL)	11.80 ± 5.71	12.92 ± 5.32	12.73 ± 4.81	13.12 ± 4.44	<0.0001
Serum Calcium (mg/dL)	9.45 ± 0.37	9.49 ± 0.37	9.47 ± 0.35	9.45 ± 0.35	0.0035
Serum Phosphorus (mg/dL)	3.80 ± 0.58	3.77 ± 0.59	3.79 ± 0.54	3.80 ± 0.56	0.4598
Total Cholesterol (mg/dL)	198.92 ± 45.99	200.77 ± 42.69	198.72 ± 40.64	202.08 ± 38.22	0.019
HDL-C (mg/dL)	50.12 ± 15.64	52.99 ± 16.34	52.71 ± 15.52	54.18 ± 15.78	<0.0001
LDL-C (mg/dL)	118.70 ± 40.69	118.67 ± 37.66	117.25 ± 36.03	118.90 ± 34.08	0.4168
Total Protein (g/L)	7.18 ± 0.47	7.15 ± 0.45	7.14 ± 0.44	7.06 ± 0.43	<0.0001
Serum Uric Acid (mg/dL)	5.37 ± 1.41	5.49 ± 1.42	5.47 ± 1.39	5.43 ± 1.32	0.0926
Serum Cotinine (ng/mL)	108.00 ± 159.75	73.85 ± 142.48	69.02 ± 136.36	46.48 ± 116.11	<0.0001
Serum Cadmium (µg/L)	0.73 ± 0.91	0.59 ± 0.66	0.55 ± 0.69	0.44 ± 0.45	<0.0001
Serum Lead (µg/L)	1.92 ± 1.69	1.83 ± 1.47	1.70 ± 1.41	1.61 ± 1.13	<0.0001
Serum Mercury (µg/L)	1.20 ± 1.73	1.40 ± 1.91	1.54 ± 2.02	2.22 ± 2.56	<0.0001
CRP (mg/L)	0.45 ± 0.85	0.40 ± 0.89	0.36 ± 0.62	0.36 ± 0.83	0.0016
BMI (Kg/m2)	28.11 ± 6.38	28.24 ± 5.79	28.18 ± 5.92	27.96 ± 5.46	0.373
Vit D (nmol/L)	58.71 ± 23.17	61.31 ± 23.74	64.31 ± 23.64	67.56 ± 22.72	<0.0001
TBS	1.37 ± 0.15	1.37 ± 0.15	1.39 ± 0.14	1.39 ± 0.14	<0.0001

Cardiovascular Diseases(%)					0.6385
Yes	2.97	3.33	2.68	2.74	
No	97.03	96.67	97.32	97.26	
Cancer(%)					0.0483
Yes	7.29	8.37	7.61	9.47	
No	92.71	91.63	92.39	90.53	
Smoking status					<0.0001
Every day	33.75	23.01	20.98	12.2	
Some days	4.92	3.45	3.46	3.19	
Not at all	17.05	26.01	23.3	28.22	
Missing	44.28	47.54	52.25	56.39	

Data are expressed as weighted means ± SE or percentages (%).

Abbreviation: SE, standard error; HDL-C, high-density lipoprotein cholesterol; LDL-C, low-density lipoprotein cholesterol; CRP, C-reactive protein; Vit D, vitamin D; BMI, body mass index; TBS, trabecular bone score.

Associations between family PIR and total TBS

Table 2 shows the regression analysis results of family PIR and TBS. In the unadjusted model, PIR was significantly positively correlated with TBS (β = 0.0046, 95% CI: 0.0026-0.0066, P < 0.001). After adjusting for covariates, models 2 and 3 still maintained this significant correlation. In the fully adjusted model (model 3), for each unit increase in PIR, TBS increased by 0.0019 (95% CI: -0.0273-0.0222, P<0.05). When grouped by income level, high-income families had a TBS higher by 0.018 compared to low-income families. Meanwhile, the trend test showed a P-value of P < 0.001, indicating that TBS significantly increases as family PIR levels rise. Moreover, in the generalized additive model and smoothing curve fitting, we found a linear relationship between PIR and TBS (Fig 2).

Fig 2. The association between family PIR and total TBS. (A) Each black point represents a sample. (B) The solid red line represents the smooth curve fit between variables. Blue bands represent the 95% of confidence interval from the fit. The model adjusts all variables except family PIR and total TBS.

Stratified analyses based on additional variables

Table 3 and Fig 3 shows the results of stratified analysis by age, gender, and race. In the unadjusted model, all age groups showed a significant positive correlation between household PIR and TBS. However, after full adjustment, only the≥50 years group maintained significance. Regarding gender, both males and females showed significant positive correlations after full adjustment. In the racial analysis, only non-Hispanic whites showed a significant association in the unadjusted model, while Mexican Americans, non-Hispanic whites, and non-Hispanic blacks all showed significant associations after full adjustment. Other racial groups showed no statistical significance before or after adjustment.

Figure 3. The association between family PIR and total TBS stratified by age (A), gender (B) and race/ethnicity (C). This model adjusted for all variables except family PIR, total TBS, and corresponding stratified variables.

Table 2. Association between the PIR and the TBS.

Variable	Model 1 β (95% CI)	Model 2 β (95% CI)	Model 3 β (95% CI)
Family PIR per 1 increase	0.0046 (0.0026, 0.0066)***	0.0064 (0.0046, 0.0082)***	0.0019 (0.0002, 0.0035)*
Low-income level (0 - 1.49)	Ref	Ref	Ref
Middle-income level (1.5 - 3.51)	-0.0058 (-0.0165, 0.0049)	0.0140 (0.0045, 0.0236)**	0.0099 (0.0021, 0.0176)*
High-income level (3.52 - 5)	0.0145 (0.0044, 0.0246)**	0.0238 (0.0147, 0.0328)***	0.0180 (0.0106, 0.0254)***
P for trend	<0.001	<0.001	<0.001

Model 1: unadjusted;

Model 2: adjusted for age, gender, and race;

Model 3: further adjusted for alkaline phosphatase, blood urea nitrogen, serum calcium, total cholesterol, serum phosphorus, serum uric acid, total protein, serum cotinine, HDL-C, LDL-C, BMI, VIT D, CRP, serum cadmium, serum lead, serum mercury, cardiovascular diseases, cancer, smoking status. P < 0.05 presents significant difference, with *p < 0.05, **p < 0.01, ***p < 0.001.

.

Table 3. Association of family PIR and TBS after stratification by age, gender, and race.

Subgroup	N	Model 1 β (95% CI)	Model 2 β (95% CI)	Model 3 β (95% CI)
Age	7832			
>=20, <35	1923	0.0065 (0.0034, 0.0096)***	0.0043 (0.0012, 0.0075)**	0.0024 (-0.0004, 0.0051)
>=35, <50	2145	0.0089 (0.0054, 0.0124)***	0.0091 (0.0055, 0.0127)***	0.0018 (-0.0012, 0.0048)
>=50	3764	0.0123 (0.0095, 0.0152)***	0.0121 (0.0092, 0.0151)***	0.0104 (0.0078, 0.0129)***
Total	7832	0.0096 (0.0078, 0.0114)***	0.0091 (0.0072, 0.0109)***	0.0055 (0.0039, 0.0071)***
Gender				
Male	3985	-0.0002 (-0.0031, 0.0026)	0.0033 (0.0006, 0.0060)*	0.0050 (0.0028, 0.0071)***
Female	3847	0.0095 (0.0067, 0.0123)***	0.0091 (0.0066, 0.0116)***	0.0038 (0.0017, 0.0060)***
Total	7832	0.0047 (0.0027, 0.0068)***	0.0064 (0.0046, 0.0082)***	0.0037 (0.0021, 0.0052)***
Race				
Mexican American	1426	0.0019 (-0.0031, 0.0069)	0.0060 (0.0015, 0.0106)**	0.0073 (0.0035, 0.0111)***
Other race/ethnicity	884	-0.0009 (-0.0064, 0.0045)	0.0033 (-0.0016, 0.0083)	0.0003 (-0.0040, 0.0045)
Non-Hispanic white	3862	0.0069 (0.0039, 0.0099)***	0.0073 (0.0047, 0.0099)***	0.0039 (0.0017, 0.0061)***
Non-Hispanic black	1660	0.0013 (-0.0033, 0.0060)	0.0031 (-0.0012, 0.0073)	0.0041 (0.0005, 0.0076)*
Total	7832	0.0052 (0.0031, 0.0072)***	0.0064 (0.0046, 0.0082)***	0.0037 (0.0021, 0.0052)***

Model 1: unadjusted.

Model 2: adjusted for age, gender, and race.

Model 3: adjusted for all variables except family PIR, total TBS, and corresponding stratified variables. P < 0.05 presents significant difference, with *p < 0.05, **p < 0.01, ***p < 0.001.

Discussion

In our study, we investigated the association between family PIR and total TBS among a large sample of the U.S. population over the age of 20. Our results showed that family PIR, whether as a continuous or categorical variable, was positively and linearly associated with total TBS when adjusted for potential confounding factors. The lower family income subgroup was younger, more often female, and more likely to belong to Non-Hispanic whites. They were also more likely to be daily smokers, suffer from vitamin D deficiency, and be exposed to environmental pollution more frequently than those in the higher family PIR category. After full adjustment, a significant association between family PIR and total TBS was found among participants aged ≥ 50, including both men and women, as well as Mexican Americans, non-Hispanic whites, and non-Hispanic blacks. This suggests that a higher family PIR is associated with a lower probability of osteoporosis and osteoporotic fractures.

Although the effect size observed in this study was small, its clinical significance remains notable. As a continuous scale measurement, the range of TBS is relatively narrow, so even minor differences may influence the diagnosis of bone microarchitecture deterioration.

Notably, this effect reflects the independent contribution of SES after adjusting for multiple factors, suggesting that the overall impact of SES on bone health may be more profound. From a population health perspective, subtle shifts in the distribution of risk factors can lead to substantial changes in disease burden. Given that SES affects a broad population, even small effects may accumulate to yield considerable public health implications for fracture prevention. The consistent associations observed across multiple subgroups in this study further affirm the clinical relevance of these findings.

Osteoporosis risk is partially determined by the cumulative effects of unhealthy dietary and activity behaviors occurring throughout life. TBS is a new complementary approach for osteoporosis evaluation in clinical practice. Several mechanisms may explain the relationship of family SES to bone health. First of all, it is likely that adults of low socioeconomic position may be at increased osteoporosis risk because of inadequate nutrition, calcium intake, and physical activity during childhood, resulting in suboptimal achievement of peak bone mass earlier in life [19-20]. Lower socioeconomic status, poverty, food insecurity, inadequate nutrition, and calcium plus vitamin D deficiency in older adulthood may result in accelerated loss of bone mass and accelerated development of

osteoporosis with age [21-22]. Evidence from a cohort study involving 246 women with osteoporosis demonstrated that 25-hydroxyvitamin D (25OHD) was associated with spine TBS, affecting bone microarchitecture and mobility performance [23]. Secondly, higher income was associated with higher self-reported physical activity [24]. Economically active individuals may engage in regular exercise, which helps improve balance and postural stability, potentially resulting in a lower risk of osteoporosis [25-26]. A cohort study from Australia involving 894 men and 682 women (ages 24-98 years) showed that lower TBS was associated with older age, increased weight, lower childhood physical activity, and lower BMD in men, and with older age, shorter stature, lower BMD, and lower mobility in women [27].

Smoking has long been identified as a risk factor for osteoporosis [28]. A cross-sectional study including 10,564 participants showed a nonlinear and positive relationship between higher serum cotinine levels (≥3 ng/ml) and the prevalence of osteoporosis [29]. Additionally, individuals in lower income jobs are more likely to carry a higher risk of occupational injury and may have workplace exposure to environmental risk factors, including hazardous wastes and toxic chemical pollution (i.e., lead), as they are more

likely to have industrial jobs [30]. This study found that serum cadmium and lead levels were significantly elevated in the low PIR group (Table 1). Heavy metal exposure may inhibit bone formation by downregulating runt-related transcription factor 2 (RUNX2) expression [31] and activate the nuclear factor kappa-B (NF-κB) pathway to promote bone resorption [32-33], thereby exacerbating the deterioration of bone microstructure. A large cross-sectional study revealed a dose-dependent relationship between TBS and bone cadmium (B-Cd) for both males and females [34].

Moreover, working individuals, citizens, and those with higher economic status were more likely to have better access to healthcare through health insurance. High-income individuals can afford the treatment and management costs of chronic diseases such as diabetes, hypertension, and osteoporosis, and take timely preventive measures and treatment [35]. In contrast, persons who lack insurance receive less medical care, including screening and treatment, and may receive poorer-quality care [36]. Compared to those with less education, well-educated respondents are more likely to be employed, earn higher incomes, and experience less economic hardship. They also tend to have extensive health knowledge, contributing to a healthier lifestyle [37]. Overall,

citizenship, working status, and economic status may serve as surrogate markers for the healthcare axis and a better lifestyle, revealing the relationship between family SES and total TBS from multiple perspectives. In summary, individuals with lower family SES may face challenges in areas such as nutritional intake, lifestyle, work-life environment, chronic disease management, and health beliefs, which can lead to osteoporosis. To clarify the relationship between family SES and total TBS, it is necessary to conduct more empirical research to understand how these factors interact and influence bone health.

Our study still has several limitations. First of all, due to its cross-sectional nature, we were unable to determine the temporal relationship between PIR and TBS. Secondly, the included covariates may not be completely accurate, which can affect the accuracy of the results. Despite these shortcomings, there are some strengths in our study, such as the fact that it is founded on a great deal of representative data from NHANES. We have demonstrated a positive association between family PIR and total TBS status. Additionally, we were able to undertake subgroup analyses of family PIR and total TBS across age, gender, and ethnicity, which has diagnostic value in predicting the presence of osteoporosis in a

patient.

Conclusion

Our results suggest that family PIR positively correlated with total TBS among U.S adults. Differences of PIR in the population should be considered in the diagnosis and treatment of osteoporosis.

Author statements

Acknowledgements

The author thanks the NHANES staff and participants for their valuable contributions.

Ethical approval

The NHANES data is publicly accessible. Per local regulations, this study was exempt from ethical review and approval.

Data availability statement

This study utilized the publicly available dataset from the NHANES in the United States (http://www.cdc.gov/nchs/nhanes/). The data were anonymized prior to analysis, participants provided informed consent, and approval was obtained from the Institutional Review Board of the National Center for Health Statistics.

Author contributions

Conceptualization: Yuan Zhang, Qice Sun, and Yanlei Li.

Data curation: Yanlei Li and Kai Chen.

Funding acquisition: Yuan Zhang and Qice Sun.

Investigation: Yuan Zhang, Tingxiao Zhao, and Qice Sun.

Methodology: Yanlei Li and Kai Chen.

Project administration: Yuan Zhang and Qice Sun.

Resources: Yuan Zhang, Tingxiao Zhao, and Qice Sun.

Software: Yanlei Li, Tingxiao Zhao, and Qice Sun.

Supervision: Yuan Zhang and Qice Sun.

Validation: Yuan Zhang, Tingxiao Zhao, and Qice Sun.

Writing-original draft: Yuan Zhang, Tingxiao Zhao, and Qice Sun.

Writing-review & editing: Yuan Zhang and Qice Sun.

References

[1] Consensus development conference: diagnosis, prophylaxis, and treatment of osteoporosis. Am J Med. 1993;94:646-650. doi:10.1016/0002-9343(93)90218-e.

[2] Kanis JA, McCloskey EV, Harvey NC, Johansson H, Leslie WD. Intervention Thresholds and the Diagnosis of Osteoporosis. J Bone Miner Res. 2015;30:1747-1753. doi:10.1002/jbmr.2531.

[3] Xu B, Radojčić MR, Anderson DB, Shi B, Yao L, Chen Y et al. Trends in prevalence of fractures among adults in the United States, 1999-2020: a population-based study. Int J Surg. 2024;110:721-732. doi: 10.1097/JS9.0000000000000883.

[4] Shen Y, Huang X, Wu J, Lin X, Zhou X, Zhu Z, et al. The Global Burden of Osteoporosis, Low Bone Mass, and Its Related Fracture in 204 Countries and Territories, 1990-2019. Front Endocrinol (Lausanne). 2022; 13:882241. doi:10.3389/fendo.2022.882241.

[5] Rosen CJ. The Epidemiology and Pathogenesis of Osteoporosis. In: Feingold KR, Ahmed SF, Anawalt B, et al., eds. Endotext. South Dartmouth (MA): MDText.com, Inc.; June 21, 2020.

[6] Pothuaud L, Carceller P, Hans D. Correlations between grey-level variations in 2D projection images (TBS) and 3D microarchitecture: applications in the study of human trabecular bone microarchitecture. Bone. 2008;42:775-787. doi:10.1016/j.bone.2007.11.018.

[7] Shevroja E, Reginster JY, Lamy O, Al-Daghri N, Chandran M, Demoux-Baiada AL, et al. Update on the clinical use of trabecular bone score (TBS) in the management of osteoporosis: results of an expert group meeting organized by the European Society for

Clinical and Economic Aspects of Osteoporosis, Osteoarthritis and Musculoskeletal Diseases (ESCEO), and the International Osteoporosis Foundation (IOF) under the auspices of WHO Collaborating Center for Epidemiology of Musculoskeletal Health and Aging. Osteoporos Int. 2023;34:1501-1529. doi:10.1007/s00198-023-06817-4.

[8] Leslie WD, Shevroja E, Johansson H, McCloskey EV, Harvey NC, Kanis JA, et al. Risk-equivalent T-score adjustment for using lumbar spine trabecular bone score (TBS): the Manitoba BMD registry. Osteoporos Int. 2018;29:751-758. doi:10.1007/s00198-018-4405-0.

[9] Shevroja E, Lamy O, Kohlmeier L, Koromani F, Rivadeneira F, Hans D. Use of Trabecular Bone Score (TBS) as a Complementary Approach to Dual-energy X-ray Absorptiometry (DXA) for Fracture Risk Assessment in Clinical Practice. J Clin Densitom. 2017;20:334-345. doi:10.1016/j.jocd.2017.06.019.

[10] Adler NE, Newman K. Socioeconomic disparities in health: pathways and policies. Health Aff (Millwood). 2002;21:60-76. doi:10.1377/hlthaff.21.2.60.

[11] Du Y, Zhao LJ, Xu Q, Wu KH, Deng HW. Socioeconomic status and bone mineral density in adults by race/ethnicity and gender: the Louisiana osteoporosis study. Osteoporos Int. 2017;28:1699-1709. doi:10.1007/s00198-017-3951-1.

[12] Brennan SL, Pasco JA, Urquhart DM, Oldenburg B, Wang Y, Wluka AE. Association between socioeconomic status and bone mineral density in adults: a systematic review. Osteoporos Int. 2011;22:517-527. doi:10.1007/s00198-010-1261-y.

[13] Xiao PL, Fuerwa C, Hsu CJ, Peng R, Cui AY, Jiang N, et al. Socioeconomic status influences on bone mineral density in American men: findings from NHANES 2011-2020.

Osteoporos Int. 2022;33:2347-2355. doi:10.1007/s00198-022-06498-5.

[13] Adler NE, Newman K. Socioeconomic disparities in health: pathways and policies. Health Aff (Millwood). 2002;21:60-76. doi:10.1377/hlthaff.21.2.60.

[14] Navarro MC, Sosa M, Saavedra P, Lainez P, Marrero M, Torres M, et al. Poverty is a risk factor for osteoporotic fractures. Osteoporos Int. 2009;20:393–398. doi: 10.1007/s00198-008-0697-9.

[15] Xiao PL, Fuerwa C, Hsu CJ, Peng R, Cui AY, Jiang N, et al. Socioeconomic status influences on bone mineral density in American men: findings from NHANES 2011-2020. Osteoporos Int. 2022;33:2347-2355. doi: 10.1007/s00198-022-06498-5.

[16] Du Y, Zhao LJ, Xu Q, Wu KH, Deng HW. Socioeconomic status and bone mineral density in adults by race/ethnicity and gender: the Louisiana osteoporosis study. Osteoporos Int. 2017;28:1699-1709. doi: 10.1007/s00198-017-3951-1.

[17] Brennan SL, Pasco JA, Urquhart DM, Oldenburg B, Hanna F, Wluka AE. The association between socioeconomic status and osteoporotic fracture in population-based adults: a systematic review. Osteoporos Int. 2009;20:1487-97. doi: 10.1007/s00198-008-0822-9.

[18] NHANES-National Health and Nutrition Examination Survey Homepage. https://www.cdc.gov/nchs/nhanes/index.htm. Published November 28, 2023. Accessed January 3, 2024.

[19] Weaver CM, Gordon CM, Janz KF, Kalkwarf HJ, Lappe JM, Lewis R, et al. The National Osteoporosis Foundation’s position statement on peak bone mass development and lifestyle factors: a systematic review and implementation recommendations. Osteoporos

Int. 2016;27:1281-1386. doi:10.1007/s00198-015-3440-3.

[20] Viljakainen HT. Factors influencing bone mass accrual: focus on nutritional aspects. Proc Nutr Soc. 2016;75:415-419. doi:10.1017/S0029665116000252.

[21] Muñoz-Garach A, García-Fontana B, Muñoz-Torres M. Nutrients and Dietary Patterns Related to Osteoporosis. Nutrients. 2020;12:1986. doi:10.3390/nu12071986.

[22] Weaver CM, Alexander DD, Boushey CJ, Dawson-Hughes B, Lappe JM, LeBoff MS, et al. Calcium plus vitamin D supplementation and risk of fractures: an updated meta-analysis from the National Osteoporosis Foundation. Osteoporos Int. 2016;27:367-376. doi:10.1007/s00198-015-3386-5.

[23] Haeri NS, Perera S, Greenspan SL. The association of vitamin D with bone microarchitecture, muscle strength, and mobility performance in older women in long-term care. Bone. 2023;176:116867. doi:10.1016/j.bone.2023.116867.

[24] Kari JT, Pehkonen J, Hirvensalo M, Yang X, Hutri-Kähönen N, Raitakari OT, et al. Income and Physical Activity among Adults: Evidence from Self-Reported and Pedometer-Based Physical Activity Measurements. Harezlak J, ed. PLoS ONE. 2015;10:e0135651. doi:10.1371/journal.pone.0135651.

[25] Kai MC, Anderson M, Lau EM. Exercise interventions: defusing the world's osteoporosis time bomb. Bull World Health Organ. 2003;81(11):827-830.

[26] Pinheiro MB, Oliveira J, Bauman A, Fairhall N, Kwok W, Sherrington C. Evidence on physical activity and osteoporosis prevention for people aged 65+ years: a systematic review to inform the WHO guidelines on physical activity and sedentary behaviour. Int J Behav Nutr Phys Act. 2020;17:150. doi:10.1186/s12966-020-01040-4.

[27] Anderson KB, Holloway-Kew KL, Hans D, Kotowicz MA, Hyde NK, Pasco JA. Physical and lifestyle factors associated with trabecular bone score values. Arch Osteoporos. 2020;15:177. doi:10.1007/s11657-020-00841-1.

[28] Cusano NE. Skeletal Effects of Smoking. Curr Osteoporos Rep. 2015;13:302-309. doi:10.1007/s11914-015-0278-8.

[29] Hou W, Chen S, Zhu C, Gu Y, Zhu L, Zhou Z. Associations between smoke exposure and osteoporosis or osteopenia in a US NHANES population of elderly individuals. Front Endocrinol (Lausanne). 2023;14:1074574. doi:10.3389/fendo.2023.1074574.

[30] Fuller R, Landrigan PJ, Balakrishnan K, Bathan G, Bose-O'Reilly S, Brauer M, et al. Pollution and health: a progress update. Lancet Planet Health. 2022;6:e535-e547. doi:10.1016/S2542-5196(22)00090-0.

[31] Jalili C, Kazemi M, Taheri E, Mohammadi H, Boozari B, Hadi A, et al. Exposure to heavy metals and the risk of osteopenia or osteoporosis: a systematic review and meta-analysis. Osteoporos Int. 2020;31:1671-1682. doi:10.1007/s00198-020-05429-6.

[32] Luo H, Gu R, Ouyang H, Wang L, Shi S, Ji Y, et al. Cadmium exposure induces osteoporosis through cellular senescence, associated with activation of NF-κB pathway and mitochondrial dysfunction. Environ Pollut. 2021;290:118043. doi: 10.1016/j.envpol.2021.

[33] Scimeca M, Feola M, Romano L, Rao C, Gasbarra E, Bonanno E, et al. Heavy metals accumulation affects bone microarchitecture in osteoporotic patients. Environ Toxicol. 2017;32:1333-1342. doi:10.1002/tox.22327.

[34] Huang JW, Fang WH, Chen WL. Detrimental Association Between Blood Cadmium

Concentration and Trabecular Bone Score. Biol Trace Elem Res. 2023;201:82-89. doi:10.1007/s12011-022-03143-4.

[35] Curtis LJ. An economic perspective on the causal explanations for the socioeconomic inequalities in health. Rev Panam Salud Publica. 2018;42:e53. doi:10.26633/RPSP.2018.53.

[36] Burstin HR, Lipsitz SR, Brennan TA. Socioeconomic status and risk for substandard medical care. JAMA. 1992;268:2383-2387.

[37] Zajacova A, Lawrence EM. The relationship between education and health: reducing disparities through a contextual approach. Annual review of public health. 2018;39:273. doi:10.1146/annurev-publhealth-031816-044628.

Dear Editors and Reviewers:

Thank you for your letter and the valuable comments from the reviewers. Based on your constructive suggestions, we have revisited and revised the manuscript. These comments provide important guidance for improving the quality of the paper. All modifications and additions have been marked in blue font. We sincerely appreciate your positive evaluation and suggestions, and hope that the revised manuscript will be accepted for publication in *PLOS ONE*.

Editor’s comments：

Thank you for submitting your manuscript to PLOS ONE. After careful consideration, we feel that it has merit but does not fully meet PLOS ONE’s publication criteria as it currently stands. Therefore, we invite you to submit a revised version of the manuscript that addresses the points raised during the review process.

Answer: Thank you for your affirmation and recognition of our article. We are fully aware that the manuscript still has shortcomings, and your professional comments are extremely valuable for improving the quality of the paper. We have made comprehensive revisions based on your suggestions and look forward to your further review and approval.

Journal Requirements:

1. Please ensure that your manuscript meets PLOS ONE's style requirements, including those for file naming. The PLOS ONE style templates can be found at https://journals.plos.org/plosone/s/file?id=wjVg/PLOSOne_formatting_sample_main_body.pdf and https://journals.plos.org/plosone/s/file?id=ba62/PLOSOne_formatting_sample_title_authors_affiliations.pdf

Answer: Thank you for your comments. This task has been completed according to your requirements.

2. We noticed you have some minor occurrence of overlapping text with the following previous publication(s), which needs to be addressed:

https://www.frontiersin.org/journals/endocrinology/articles/10.3389/fendo.2022.857110/full
https://pubmed.ncbi.nlm.nih.gov/36626464/
https://link.springer.com/article/10.1007/s00198-005-1917-1?

In your revision ensure you cite all your sources (including your own works), and quote or rephrase any duplicated text outside the methods section. Further consideration is dependent on these concerns being addressed.

Answer: Thank you for your comments. Your suggestions are very important for improving the quality of the manuscript. We sincerely apologize for the text overlap issue and have thoroughly revised the manuscript.

3. Please note that your Data Availability Statement is currently missing the DOI/accession number of each dataset or a direct link to access each database. If your manuscript is accepted for publication, you will be asked to provide these details on a very short timeline. We therefore suggest that you

provide this information now, though we will not hold up the peer review process if you are unable.

Answer: Thank you for your comments. Your suggestions are very important for improving the quality of the manuscript. We have addressed your comments and completed the task as per your request.

4. PLOS requires an ORCID iD for the corresponding author in Editorial Manager on papers submitted after December 6th, 2016. Please ensure that you have an ORCID iD and that it is validated in Editorial Manager. To do this, go to ‘Update my Information’ (in the upper left-hand corner of the main menu), and click on the Fetch/Validate link next to the ORCID field. This will take you to the ORCID site and allow you to create a new iD or authenticate a pre-existing iD in Editorial Manager.

Answer: Thank you for your comments. This task has been completed according to your requirements.

5. Please include a separate caption for each figure in your manuscript.

Answer: Thank you for your comments. This task has been completed according to your requirements.

Additional Editor Comments:

Ensure all abbreviations are defined at first use.

Answer: Thank you for your comments. This task has been completed according to your requirements.

Introduction

Change “This study addresses the gap in ...general population”

to "This study aims to address the gap in ... general population.”

Answer: Thank you for your comments. Your suggestions are very important for improving the quality of the manuscript. We have addressed your comments and completed the task as per your request.

Fig 1 should be part of the methods. Remove arrows from figure. Use neutral connectors instead.

Answer: Thank you for your comments. Your suggestions are very important for improving the quality of the manuscript. Arrows, as internationally recognized symbols in flowcharts, are widely used in academic journal flowcharts. They visually demonstrate the sequence and direction of screening steps, conforming to international guideline standards and readers' reading habits, thereby enhancing the readability and academic quality of diagrams.

Methods

Provide the rationale for the selection of confounders and the choice statistical methods, especially for stratified and interaction analyses.

Answer: We thank the reviewer for this comment. We have added a clear rationale for our selection of confounders and statistical methods in the Methods section.

Results

Table 3 is too crowded; please divide it into two separate tables to improve clarity.

Answer: Thank you for your comments. Your suggestions are very important for improving the quality of the manuscript. We have addressed your comments and completed the task as per your request.

Discussion

Discuss the clinical significance of the small observed effect size?

Answer: Thank you for your comments. We acknowledge the importance of addressing the clinical significance of the observed effect size. We have added a new paragraph in the Discussion section to specifically address this point.

Discuss possible mechanisms linking SES and TBS.

Answer: Thank you for your comments. Your suggestions are very important for improving the quality of the manuscript. We have addressed your comments and completed the task as per your request.

Explicitly mention study limitations,....use "temporal" instead of "causal"

Answer: Thank you for your comments. This task has been completed according to your requirements.

Ethics

Add a brief statement clarifying that no ethics approval was needed due to the use of publicly available, de-identified NHANES data. Please check journal's guidelines

Answer: Thank you for your comments. This task has been completed according to your requirements.

Comments to the Author

1. Is the manuscript technically sound, and do the data support the conclusions?

The manuscript must describe a technically sound piece of scientific research with data that supports the conclusions. Experiments must have been conducted rigorously, with appropriate controls, replication, and sample sizes. The conclusions must be drawn appropriately based on the data presented.

Reviewer #1: Yes

Answer: Thank you for your comments. We appreciate your positive remarks on our manuscript. We sincerely hope that our article will be accepted for publication.

---

2. Has the statistical analysis been performed appropriately and rigorously?

Reviewer #1: Yes

Answer: Thank you for your comments. We appreciate your positive remarks on our manuscript. We sincerely hope that our article will be accepted for publication.

---

3. Have the authors made all data underlying the findings in their manuscript fully available?

The PLOS Data policy requires authors to make all data underlying the findings described in their manuscript fully available without restriction, with rare exception (please refer to the Data Availability Statement in the manuscript PDF file). The data should be provided as part of the manuscript or its supporting information, or deposited to a public repository. For example, in addition to summary statistics, the data points behind means, medians and variance measures should be available. If there are restrictions on publicly sharing data—e.g. participant privacy or use of data from a third party—those must be specified.

Reviewer #1: Yes

Answer: Thank you for your comments. We appreciate your positive remarks on our manuscript. We sincerely hope that our article will be accepted for publication.

---

4. Is the manuscript presented in an intelligible fashion and written in standard English?

PLOS ONE does not copyedit accepted manuscripts, so the language in submitted articles must be clear, correct, and unambiguous. Any typographical

or grammatical errors should be corrected at revision, so please note any specific errors here.

Reviewer #1: Yes

Answer: Thank you for your comments. We appreciate your positive remarks on our manuscript. We sincerely hope that our article will be accepted for publication.

---

6. Review Comments to the Author

Please use the space provided to explain your answers to the questions above. You may also include additional comments for the author, including concerns about dual publication, research ethics, or publication ethics. (Please upload your review as an attachment if it exceeds 20,000 characters)

Answer: Thank you again for your positive and constructive comments and suggestions on our manuscript. We hope you will find our revised manuscript acceptable for publication.

Reviewer #1: I don’t have a lot of concerns about this study – large sample size and an unsurprising finding demonstrating a relationship between SES and TBS. There are these three things I suggest the authors correct, including:

Answer: We feel great thanks for your professional review work on our article. As you are concerned, there are several problems that need to be addressed. According to your nice suggestions, we have made extensive corrections to our previous draft, the detailed corrections are listed below.

1. Introduction needs a hypothesis.

Answer: Thank you for your comments. Your suggestions are very important for improving the quality of the manuscript. We have addressed your comments and completed the task as per your request.

2. Introduction needs expanding to include literature about other studies have looked at the relationship between SES and osteoporosis/bone health. There are plenty of them and the readers will need to get a good picture of what has already been done in this space, before understanding that there is a need to look at TBS. In fact, there have been studies that found inconsistent relationships between SES and different measures of bone remodelling/BMD

etc. It will be important to establish a good overview of the current state of the literature there.

Answer: Thank you for your comments. Your suggestions are very important for improving the quality of the manuscript. We have addressed your comments and completed the task as per your request.

3. In Figures 2 and 3, the font on the x and y axes and in the legend, is too small.

Answer: Thank you for your comments. This task has been completed according to your requirements.

---

6. PLOS authors have the option to publish the peer review history of their article (what does this mean?). If published, this will include your full peer review and any attached files.

If you choose “no”, your identity will remain anonymous but your review may still be made public.

Do you want your identity to be public for this peer review? For information about this choice, including consent withdrawal, please see our Privacy Policy.

Reviewer #1: No

We sincerely thank the editor and all reviewers for their valuable feedback that we have used to improve the quality of our manuscript. We hope our revisions meet your approval and contribute meaningful insights to the field. We welcome any further comments or suggestions.

Yours sincerely

Qice Sun

July 15, 2025
