# Supplementary material for: Unveiling the wealth-bone connection: How socioeconomic status influences trabecular bone health
Source: PLoS One. 2025 Dec 12;20(12):e0338378. doi: 10.1371/journal.pone.0338378 (PMC12700419; doi:10.1371/journal.pone.0338378)
Supplement: Attachment — Submitted filename: Response to reviewer-PLOS ONE.docx [file pone.0338378.s001.docx]

Dear Editors and Reviewers:

Thank you for your letter and the valuable comments from the reviewers. Based on your constructive suggestions, we have revisited and revised the manuscript. These comments provide important guidance for improving the quality of the paper. All modifications and additions have been marked in blue font. We sincerely appreciate your positive evaluation and suggestions, and

hope that the revised manuscript will be accepted for publication in *PLOS ONE*.

**Editor’s comments：**

Thank you for submitting your manuscript to PLOS ONE. After careful consideration, we feel that it has merit but does not fully meet PLOS ONE’s publication criteria as it currently stands. Therefore, we invite you to submit a revised version of the manuscript that addresses the points raised during the review process.

Answer: Thank you for your affirmation and recognition of our article. We are fully aware that the manuscript still has shortcomings, and your professional comments are extremely valuable for improving the quality of the paper. We have made comprehensive revisions based on your suggestions and look forward to your further review and approval.

**Journal Requirements:**

1. Please ensure that your manuscript meets PLOS ONE's style requirements, including those for file naming. The PLOS ONE style templates can be found at [https://journals.plos.org/plosone/s/file?id=wjVg/PLOSOne_formatting_sample_main_body.pdf](https://journals.plos.org/plosone/s/file?id=wjVg/PLOSOne_formatting_sample_main_body.pdf) and
   [https://journals.plos.org/plosone/s/file?id=ba62/PLOSOne_formatting_sample_title_authors_affiliations.pdf](https://journals.plos.org/plosone/s/file?id=ba62/PLOSOne_formatting_sample_title_authors_affiliations.pdf)

Answer: Thank you for your comments. This task has been completed according to your requirements.

2. We noticed you have some minor occurrence of overlapping text with the following previous publication(s), which needs to be addressed:

[https://www.frontiersin.org/journals/endocrinology/articles/10.3389/fendo.2022.857110/full](https://www.frontiersin.org/journals/endocrinology/articles/10.3389/fendo.2022.857110/full)
[https://pubmed.ncbi.nlm.nih.gov/36626464/](https://pubmed.ncbi.nlm.nih.gov/36626464/)
[https://link.springer.com/article/10.1007/s00198-005-1917-1?](https://link.springer.com/article/10.1007/s00198-005-1917-1?)

In your revision ensure you cite all your sources (including your own works), and quote or rephrase any duplicated text outside the methods section. Further consideration is dependent on these concerns being addressed.

Answer: Thank you for your comments. Your suggestions are very important for improving the quality of the manuscript. We sincerely apologize for the text overlap issue and have thoroughly revised the manuscript.

3. Please note that your Data Availability Statement is currently missing the DOI/accession number of each dataset or a direct link to access each database. If your manuscript is accepted for publication, you will be asked to provide these details on a very short timeline. We therefore suggest that you provide this information now, though we will not hold up the peer review process if you are unable.

Answer: Thank you for your comments. Your suggestions are very important for improving the quality of the manuscript. We have addressed your comments and completed the task as per your request.

4. PLOS requires an ORCID iD for the corresponding author in Editorial Manager on papers submitted after December 6th, 2016. Please ensure that you have an ORCID iD and that it is validated in Editorial Manager. To do this, go to ‘Update my Information’ (in the upper left-hand corner of the main menu), and click on the Fetch/Validate link next to the ORCID field. This will take you to the ORCID site and allow you to create a new iD or authenticate a pre-existing iD in Editorial Manager.

Answer: Thank you for your comments. This task has been completed according to your requirements.

5. Please include a separate caption for each figure in your manuscript.

Answer: Thank you for your comments. This task has been completed according to your requirements.

**Additional Editor Comments:**
Ensure all abbreviations are defined at first use.
Answer: Thank you for your comments. This task has been completed according to your requirements.

Introduction
Change “This study addresses the gap in ...general population”
to "This study aims to address the gap in ... general population.”

Answer: Thank you for your comments. Your suggestions are very important for improving the quality of the manuscript. We have addressed your comments and completed the task as per your request.

Fig 1 should be part of the methods. Remove arrows from figure. Use neutral connectors instead.
Answer: Thank you for your comments. Your suggestions are very important for improving the quality of the manuscript. Arrows, as internationally recognized symbols in flowcharts, are widely used in academic journal flowcharts. They visually demonstrate the sequence and direction of screening steps, conforming to international guideline standards and readers' reading habits, thereby enhancing the readability and academic quality of diagrams.

Methods
Provide the rationale for the selection of confounders and the choice statistical methods, especially for stratified and interaction analyses.

Answer: We thank the reviewer for this comment. We have added a clear rationale for our selection of confounders and statistical methods in the Methods section.

Results
Table 3 is too crowded; please divide it into two separate tables to improve clarity.

Answer: Thank you for your comments. Your suggestions are very important for improving the quality of the manuscript. We have addressed your comments and completed the task as per your request.

Discussion
Discuss the clinical significance of the small observed effect size?

Answer: Thank you for your comments. We acknowledge the importance of addressing the clinical significance of the observed effect size. We have added a new paragraph in the Discussion section to specifically address this point.

Discuss possible mechanisms linking SES and TBS.

Answer: Thank you for your comments. Your suggestions are very important for improving the quality of the manuscript. We have addressed your comments and completed the task as per your request.

Explicitly mention study limitations,....use "temporal" instead of "causal"
Answer: Thank you for your comments. This task has been completed according to your requirements.

Ethics
Add a brief statement clarifying that no ethics approval was needed due to the use of publicly available, de-identified NHANES data. Please check journal's guidelines

Answer: Thank you for your comments. This task has been completed according to your requirements.

**Comments to the Author**
1. Is the manuscript technically sound, and do the data support the conclusions?
The manuscript must describe a technically sound piece of scientific research with data that supports the conclusions. Experiments must have been conducted rigorously, with appropriate controls, replication, and sample sizes. The conclusions must be drawn appropriately based on the data presented.

Reviewer #1: Yes

Answer: Thank you for your comments. We appreciate your positive remarks on our manuscript. We sincerely hope that our article will be accepted for publication.

2. Has the statistical analysis been performed appropriately and rigorously?

Reviewer #1: Yes

Answer: Thank you for your comments. We appreciate your positive remarks on our manuscript. We sincerely hope that our article will be accepted for publication.

3. Have the authors made all data underlying the findings in their manuscript fully available?

The [PLOS Data policy](http://track.editorialmanager.com/CL0/http://www.plosone.org/static/policies.action%23sharing/1/010f01979cf48223-3a6a7db4-d99c-4c45-85da-323e3930bf62-000000/O8UFeW5JjqlsCf-x05FfuYkkhK5xL6brslwgyQvBDNg=216) requires authors to make all data underlying the findings described in their manuscript fully available without restriction, with rare exception (please refer to the Data Availability Statement in the manuscript PDF file). The data should be provided as part of the manuscript or its supporting information, or deposited to a public repository. For example, in addition to summary statistics, the data points behind means, medians and variance measures should be available. If there are restrictions on publicly sharing data—e.g. participant privacy or use of data from a third party—those must be specified.

Reviewer #1: Yes

Answer: Thank you for your comments. We appreciate your positive remarks on our manuscript. We sincerely hope that our article will be accepted for publication.

4. Is the manuscript presented in an intelligible fashion and written in standard English?
PLOS ONE does not copyedit accepted manuscripts, so the language in submitted articles must be clear, correct, and unambiguous. Any typographical or grammatical errors should be corrected at revision, so please note any specific errors here.

Reviewer #1: Yes

Answer: Thank you for your comments. We appreciate your positive remarks on our manuscript. We sincerely hope that our article will be accepted for publication.

6. Review Comments to the Author

   Please use the space provided to explain your answers to the questions above. You may also include additional comments for the author, including concerns about dual publication, research ethics, or publication ethics. (Please upload your review as an attachment if it exceeds 20,000 characters)

Answer: Thank you again for your positive and constructive comments and suggestions on our manuscript. We hope you will find our revised manuscript acceptable for publication.

Reviewer #1: I don’t have a lot of concerns about this study – large sample size and an unsurprising finding demonstrating a relationship between SES and TBS. There are these three things I suggest the authors correct, including:

 Answer: We feel great thanks for your professional review work on our article. As you are concerned, there are several problems that need to be addressed. According to your nice suggestions, we have made extensive corrections to our previous draft, the detailed corrections are listed below.

1. Introduction needs a hypothesis.

Answer: Thank you for your comments. Your suggestions are very important for improving the quality of the manuscript. We have addressed your comments and completed the task as per your request.

2. Introduction needs expanding to include literature about other studies have looked at the relationship between SES and osteoporosis/bone health. There are plenty of them and the readers will need to get a good picture of what has already been done in this space, before understanding that there is a need to look at TBS. In fact, there have been studies that found inconsistent relationships between SES and different measures of bone remodelling/BMD etc. It will be important to establish a good overview of the current state of the literature there.

Answer: Thank you for your comments. Your suggestions are very important for improving the quality of the manuscript. We have addressed your comments and completed the task as per your request.

3. In Figures 2 and 3, the font on the x and y axes and in the legend, is too small.

Answer: Thank you for your comments. This task has been completed according to your requirements.

6. PLOS authors have the option to publish the peer review history of their article ([what does this mean?](https://track.editorialmanager.com/CL0/https://journals.plos.org/plosone/s/editorial-and-peer-review-process%23loc-peer-review-history/1/010f01979cf48223-3a6a7db4-d99c-4c45-85da-323e3930bf62-000000/xJAn4-1qpeQLK68HmRBpHzEf3c96CQqqbVxN-PzlBbw=216)). If published, this will include your full peer review and any attached files.
If you choose “no”, your identity will remain anonymous but your review may still be made public.
Do you want your identity to be public for this peer review? For information about this choice, including consent withdrawal, please see our [Privacy Policy](https://track.editorialmanager.com/CL0/https://www.plos.org/privacy-policy/1/010f01979cf48223-3a6a7db4-d99c-4c45-85da-323e3930bf62-000000/7JpoZ5qSgBfL2n0QC_pp0PdghsbknxA7vKcRuoU4CR0=216).

Reviewer #1: No

We sincerely thank the editor and all reviewers for their valuable feedback that we have used to improve the quality of our manuscript. We hope our revisions meet your approval and contribute meaningful insights to the field. We welcome any further comments or suggestions.

Yours sincerely

Qice Sun

July 15, 2025
