# Supplementary material for: Unveiling the wealth-bone connection: How socioeconomic status influences trabecular bone health
Source: PLoS One. 2025 Dec 12;20(12):e0338378. doi: 10.1371/journal.pone.0338378 (PMC12700419; doi:10.1371/journal.pone.0338378)
Supplement: Attachment — Submitted filename: Response to Reviewers.docx [file pone.0338378.s003.docx]

**To:**
The Editorial Office
PLOS ONE

**Subject:** Revised Submission of Manuscript PONE-D-24-55944R1
**Title:** Unveiling the Wealth-Bone Connection: How Socioeconomic Status Influences Trabecular Bone Health

**Dear Editor,**

We sincerely thank you and the reviewer for your constructive and thoughtful feedback on our manuscript. We are pleased that Reviewer #1 found our previous revisions satisfactory and considered the manuscript much improved.

In accordance with the reviewer’s remaining suggestions, we have made several minor textual revisions to improve clarity and cultural sensitivity. Specifically, we replaced terms such as race, blacks, and whites with more culturally appropriate and scientifically precise expressions including ethnicity, ancestry, African Americans, and Caucasians. These changes have been carefully applied throughout the Methods, Results, and Discussion sections.

We have also reviewed the reference list for completeness and accuracy, ensuring compliance with the PLOS ONE reference and data availability policies.

A detailed, point-by-point rebuttal addressing the reviewer’s comments is included as a separate document, and all textual changes have been highlighted in the revised manuscript for ease of review.

We thank the reviewers and editorial team again for their time and effort in improving the quality of our work. We believe that the revised version of our manuscript now meets the journal’s publication standards, and we respectfully submit it for your further consideration.

**Sincerely,**
Qice Sun
The Second Affiliated Hospital of Zhejiang Chinese Medical University
xinhuasunqice@163.com

## ****Response to Review Comments****

**Manuscript Title:** Unveiling the Wealth-Bone Connection: How Socioeconomic Status Influences Trabecular Bone Health
**Manuscript ID:** PONE-D-24-55944R1

**Dear Editor,**

We sincerely thank you and the reviewer for the positive evaluation of our revised manuscript. We are pleased that Reviewer #1 found our responses satisfactory and considered the manuscript much improved.

Below, we provide our responses to the minor remaining suggestions.

### ****Editorial Requirements****

We have also reviewed the reference list to ensure it is accurate and that no retracted works are cited. All data remain publicly available in accordance with the PLOS Data Policy.

Journal Requirements:

If the reviewer comments include a recommendation to cite specific previously published works, please review and evaluate these publications to determine whether they are relevant and should be cited. There is no requirement to cite these works unless the editor has indicated otherwise. 
Response：Thank you for your attention. The reviewer's comments did not include suggestions for citing published literature.

2. Please review your reference list to ensure that it is complete and correct. If you have cited papers that have been retracted, please include the rationale for doing so in the manuscript text, or remove these references and replace them with relevant current references. Any changes to the reference list should be mentioned in the rebuttal letter that accompanies your revised manuscript. If you need to cite a retracted article, indicate the article’s retracted status in the References list and also include a citation and full reference for the retraction notice.

Response：Thank you for your attention. We carefully checked the list of references to ensure that the content was complete and accurate, and that there were no papers that had been retracted.

**Reviewers' comments:**

Reviewer's Responses to Questions

**Comments to the Author**

1. If the authors have adequately addressed your comments raised in a previous round of review and you feel that this manuscript is now acceptable for publication, you may indicate that here to bypass the “Comments to the Author” section, enter your conflict of interest statement in the “Confidential to Editor” section, and submit your "Accept" recommendation.

Reviewer #1: All comments have been addressed

Response: Thank you for your attention.

2. Is the manuscript technically sound, and do the data support the conclusions?

The manuscript must describe a technically sound piece of scientific research with data that supports the conclusions. Experiments must have been conducted rigorously, with appropriate controls, replication, and sample sizes. The conclusions must be drawn appropriately based on the data presented.

Reviewer #1: Yes

Response: Thank you for your attention.

3. Has the statistical analysis been performed appropriately and rigorously?

Reviewer #1: Yes

Response: Thank you for your attention.

4. Have the authors made all data underlying the findings in their manuscript fully available?

The [PLOS Data policy](http://track.editorialmanager.com/CL0/http://www.plosone.org/static/policies.action%23sharing/1/010f0199c6b77132-4d315060-8457-493c-b865-6f303825dbed-000000/S7n55_MW36BENeJcPQLD_Fsss7G6FdPKwhFV8xJ7g7Y=232) requires authors to make all data underlying the findings described in their manuscript fully available without restriction, with rare exception (please refer to the Data Availability Statement in the manuscript PDF file). The data should be provided as part of the manuscript or its supporting information, or deposited to a public repository. For example, in addition to summary statistics, the data points behind means, medians and variance measures should be available. If there are restrictions on publicly sharing data—e.g. participant privacy or use of data from a third party—those must be specified.

Reviewer #1: Yes

Response: Thank you for your attention.

5. Is the manuscript presented in an intelligible fashion and written in standard English?

PLOS ONE does not copyedit accepted manuscripts, so the language in submitted articles must be clear, correct, and unambiguous. Any typographical or grammatical errors should be corrected at revision, so please note any specific errors here.

Reviewer #1: Yes

Response: Thank you for your attention.

6. Review Comments to the Author

Please use the space provided to explain your answers to the questions above. You may also include additional comments for the author, including concerns about dual publication, research ethics, or publication ethics. (Please upload your review as an attachment if it exceeds 20,000 characters)

Reviewer #1: This is a much-improved manuscript. I have made some very minor comments in text on the writing mechanics here and there - please see file attached. I also suggest to rephrase the elements of the text that refer to the 'racial' analysis - it would be more culturally sensitive to use terms such as ethnicity or ancestry rather than race. Same goes for the use of 'blacks' 'whites' etc. - please consider using terms such as African-Americans, Caucasians etc.

Response: We thank the reviewer for this valuable suggestion. Following this advice, we have carefully revised the manuscript to replace all terms referring to “race” with more culturally appropriate and scientifically precise terminology. Specifically:

“race” has been replaced by “ethnicity” or “ancestry,” depending on context.

“blacks” and “whites” have been replaced with “African Americans” and “Caucasians,” respectively.

All corresponding sentences have been reviewed to ensure consistent and respectful language throughout the manuscript.

These changes have been made in the Methods, Results, and Discussion sections accordingly (highlighted in the revised version).

7. PLOS authors have the option to publish the peer review history of their article ([what does this mean?](https://track.editorialmanager.com/CL0/https://journals.plos.org/plosone/s/editorial-and-peer-review-process%23loc-peer-review-history/1/010f0199c6b77132-4d315060-8457-493c-b865-6f303825dbed-000000/M73dPeeWWSLvE8kTPEnpJdhT9JLhEaqvgyOn1Qe4FqA=232)). If published, this will include your full peer review and any attached files.


If you choose “no”, your identity will remain anonymous but your review may still be made public.


**Do you want your identity to be public for this peer review?** For information about this choice, including consent withdrawal, please see our [Privacy Policy](https://track.editorialmanager.com/CL0/https://www.plos.org/privacy-policy/1/010f0199c6b77132-4d315060-8457-493c-b865-6f303825dbed-000000/wAdCQMKvMNN5QH7gCthMi2WGWbWI3Ijej1BiyddnILA=232).

Reviewer #1: No

Response: Thank you for your attention.
